# Cryptic Cryptophytes—Revision of the Genus *Goniomonas*

**DOI:** 10.1111/jeu.70038

**Published:** 2025-09-12

**Authors:** Maria Sachs, Frank Nitsche, Hartmut Arndt

**Affiliations:** ^1^ Institute of Zoology University of Cologne Cologne Germany; ^2^ Senckenberg am Meer, DZMB – German Centre for Marine Biodiversity Research Wilhelmshaven Germany

**Keywords:** Cryptista, Cryptophyta, heterotrophic flagellates, phylogeny, salinity, synapomorphy

## Abstract

Cryptomonad protists are ubiquitously distributed over marine and freshwater habitats. As an exception to the colored cryptomonads, the heterotrophic cryptomonads of the genus *Goniomonas* have an ancestral phylogenetic position. They lack any kind of chloroplast and most likely represent a basal group to those cryptomonad groups having obtained their chloroplast by secondary endosymbiosis. Earlier studies have shown a deep divergence between freshwater and marine clades of goniomonads that comprise large genetic distances between members within the group and also between the two groups of marine and freshwater taxa. Still, marine and freshwater species carry the same genus name, and to date, only a few species have been described. We therefore restructured goniomonad systematics based not only on a separation of marine and freshwater taxa, but also, taking the large genetic distances into account, on several new genera that are described. Based on morphological as well as phylogenetic data (18S rDNA sequences), this leads to the formation of the freshwater genera *Limnogoniomonas* n. g., *Goniomonas*, and *Aquagoniomonas* n. g. and the marine genera *Neptunogoniomonas* n. g., *Baltigoniomonas* n. g., *Marigoniomonas* n. g., *Thalassogoniomonas* n. g., *Poseidogoniomonas* n. g., and *Cosmogoniomonas* n. g. To give the restructuring process a stable basis, we additionally propose a neotype for *Goniomonas truncata*.

## 1 Introduction

Cryptomonad flagellates are small, mostly bean‐shaped, non‐colonial protists that encompass at least 200 species (Novarino 2003). The group is globally distributed in marine, brackish, and freshwater habitats (Archibald 2020). Cryptomonads played a crucial role in understanding evolutionary processes, as phototrophic representatives are the outcome of secondary endosymbiosis (Cenci et al. 2018; McFadden 2018). Phototrophic cryptomonads had engulfed red algae, which themselves were originally created through the engulfment of a cyanobacterium by a eukaryotic phagotroph during primary endosymbiosis (Keeling 2004). To understand the evolutionary history of cryptophytes and elucidate their unique characteristics and adaptations, Douglas and Penny (1999) provided important insights into the evolutionary dynamics of their plastid genomes by emphasizing the descent of their plastids from red algae and the retention of nucleomorphs as a distinguishing feature among secondary plastid‐bearing algae (Douglas and Penny 1999). Within the larger group of cryptists, which includes *Palpitomonas bilix* and kathablepharids (as evolutionary more basal groups), as well as cryptophytes and goniomonads, it is a likely scenario that photosynthesis was established after the split of the latter two (Yabuki et al. 2014). This would potentially make goniomonads the last form of pre‐endosymbionts before the engulfment of plastids. Cenci et al. (2018) sequenced the entire genome of the most recently described goniomonad species, *Goniomonas avonlea*. On a genomic or phylogenetic level, no convincing evidence could be found that *G. avonlea* ever possessed a plastid, supporting the theory that *Goniomonas* is indeed a very basal phagotroph, a “pre‐secondary‐endosymbiosis cryptomonad” (McFadden 2018). Apart from the lack of plastids, all goniomonads share a number of morphological traits, such as a laterally flattened, anteriorly more or less truncated cell body and a horizontal row of ejectosomes in the anterior part of the cell (Novarino 2003). Unlike other cryptomonads, which were reported to have a “recoiling” swimming behavior (Novarino 2003), goniomonads display a rather “jerky” movement (Kim and Archibald 2013) and move on the substrate along irregular tracks while rotating. When analyzing sediment‐associated protists, *Goniomonas*‐like flagellates are among the 20 most commonly observed heterotrophic flagellate morphotypes (Patterson and Lee 2000) and have been recorded in many different habitats (Arndt et al. 2000). Until the taxon was transferred to the currently used named genus *Goniomonas* by Stein (1878), it was considered under the protonym *Monas truncata* (Fresenius 1858). Larsen and Patterson (1990) discussed the history of using the genus name in the past and described two new marine species of *Goniomonas*. Up to now, five different species have been described, with *G. avonlea* (Kim and Archibald 2013) being the most recent. Apart from the type species *Goniomonas truncata* and *Goniomonas brasiliensis* (Jarreta de Castro and Bicudo 2023) which were isolated from freshwater, the three other species are marine species: *Goniomonas amphinema*, *Goniomonas pacifica* (see Larsen and Patterson 1990) and *G. avonlea* (Kim and Archibald 2013).

Recently, the parallel study of Phanprasert et al. (2025) added another three marine species, *G*. *ulleungensis, G. lingua*, and *G. duplex*, to the list of scientifically described species. Molecular phylogenetic analyses revealed a deep divergence between the goniomonad species from marine and freshwaters (Von Der Heyden et al. 2004). Studies on other protist groups have shown similar differences in ecological clusters associated with molecular differentiations, for example, the choanoflagellate taxa *Codosiga* and *Hartaetosiga* (Carr et al. 2017). The high divergence among the genus *Goniomonas* could imply that there are still multiple undescribed species and that the genus is generally under‐split from a taxonomical point of view (Von Der Heyden et al. 2004). The taxonomic situation becomes even more complex since type material other than drafts of microscopic images is missing. Studies by Shalchian‐Tabrizi et al. (2008) and Shiratori and Ishida (2016) have already indicated a large hidden diversity within cryptomonads, including heterotrophic lineages.

In this study, we describe seven new species and emend several other strains from different aquatic habitats including, marine, brackish, and freshwater environments, and propose the revision of the genus *Goniomonas* into *Goniomonas* sensu strictu, which includes freshwater species and a newly created neotype for *Goniomonas truncata*, as well as five new genera within both the freshwater and marine clusters based mainly on genetic distances, including also the recently described species of Phanprasert et al. (2025). Morphological descriptions are supported by light and electron micrographs and genetic divergences based on 18S rDNA phylogeny.

## 2 Materials and Methods

Sediment and plankton samples were taken at diverse sampling sites (Table S8). Sediment samples were taken with a Multi‐Corer system (MUC), while plankton samples were either taken with a CTD‐rosette system from different depths or were taken with flasks from surface waters. Subsamples of a few milliliters were suspended in ambient autoclaved water (approx. 20 mL) and transferred to 50‐mL tissue culture flasks (Sarstedt, Nümbrecht, Germany). Isolation of cells from raw cultures was carried out by micromanipulation under an inverted microscope or by the liquid aliquot method, pipetting a few microliters of raw cultures in either 0.8‐ or 0.4‐mL multi‐well plates containing ambient autoclaved water. Culture flasks and wells were supplied with autoclaved quinoa grains to support the growth of autochthonous bacteria.

Mono‐cultures of strains HFCC (**H**eterotrophic **F**lagellate Culture **C**ollection **C**ologne at the University of Cologne) HFCC22, HFCC157, HFCC272, HFCC841, and HFCC1666 with marine/brackish origin were further cultivated in Schmalz‐Pratt medium (Table S1). Salinity was adjusted to the original salinity of the sampling site. Freshwater strains HFCC235, HFCC251, HFCC23, HFCC5007, and HFCC162 were cultivated in WC medium (Guillard and Lorenzen 1972). To stimulate the growth of associated bacteria, either autoclaved wheat or quinoa grains were added to the cultures as a carbon source.

### 2.1 Morphological Analyses

Light microscopical investigations were carried out using an inverted microscope (Zeiss Axio Observer; water immersion condenser; 100×/1.4NA oil immersion objective and differential interference contrast). High resolution videos were recorded from cultures in Petri dishes with coverslips using two ways. Earlier records were based on an Allen Video‐enhanced system (Hamamatsu C6489, Argus‐20) to suppress noise and amplify contrast. Frame‐by‐frame evaluation of videos was carried out using VirtualDub (www.virtualdub.org), and editing was done by ImageJ (Abramoff et al. 2004). Morphological measurements were carried out using Axio Vision Rel. 4.8 (Zeiss, Germany). Observations of recent isolates were made using videos recorded with the camera Axiocam 305 mono 31,812 (Zeiss, Germany). Pictures were analyzed using the ZEISS ZEN 3.5 blue edition program.

For electron microscopical investigations, cultures were fixed with 0.1 M cacodylate buffered glutaraldehyde solution (2.5%) for one hour at 4°C. Fixed cells were washed twice with cacodylate buffer and once with distilled water and then stained with 0.5% osmium tetroxide solution for 10 min. After two additional washing steps with distilled water, the cells were stepwise dehydrated with an increasing ethanol series (30%, 50%, 70%, 80%, 90%, 96%; each step 15 min) followed by ethanol/HMDS (1:1) and dried in pure HDMS (Hexamethyldisilazan, Carl Roth, Germany). Before examination by SEM (Fei Quanta 250 FEG at the Biocenter, University of Cologne), cells were sputtered with a gold layer (12 nm).

### 2.2 Molecular Studies

Molecular analyses followed procedures described by Schoenle et al. (2020). Strains were centrifuged (4000 g for 20 min at 4°C, Megafuge 2.0 R, Heraeus Instruments), and genomic DNA was extracted using the Quick‐gDNA MiniPrep (Zymo Research, USA). For sequencing partial SSU rDNA, three different sets of primers were used: 18S‐For (5′‐AACCTGGTTGATCCTGCCAGT 3′) and 18S‐Rev (5′TGATCCTTCTGCAGGTTCACCTAC 3′) (Medlin et al. 1988), 590For (5′‐CGGTAATTCCAGCTCCAATAGC‐3′) in combination with 1300 Rev (5′‐CACCAACTAAGAACGGCCATGC‐‘3) (Wylezich et al. 2002), and the set 82F (5’‐GAAACTGCGAATGGCTC‐3′) (López‐García et al. 2002) in combination with Rev‐1 (5′‐ACCTACGGAAACCTTGTTACG‐3′) (newly assigned) were used for amplification with a primer concentration of 1 μM and the help of a PCR Mastermix (VWR Life Science, Red Taq DNA Polymerase, Hassrode, Belgium). For the first two primer sets, the following steps were applied: an initial denaturation step at 98°C for 2 min, followed by 35 cycles of 98°C for 30 s, 55°C for 45 s, and 72°C for 2.30 min, and a final elongation step for 10 min at 72°C. For the third primer set, the following steps were used: initial denaturation at 98°C for 2 min, 35 cycles at 98°C for 30 s, at 52°C for 45 s, at 48°C for 30 s, and at 72°C for 2.30 min, a final elongation step at 72°C for 10 min. The length of PCR products was checked on a 1% agarose gel and subsequently purified with the PCR Purification Kit (Jena Bioscience, Jena, Germany). Purified PCR products were sequenced at GATC Biotech, Germany.

### 2.3 Phylogenetic Analyses

The 18S rDNA of the 26 new goniomonad sequences was aligned with 25 closely related *Goniomonas* sequences from GenBank and the three most recently described strains by Phanprasert et al. (2025), as well as six outgroup sequences of the CRY1 group (following Shiratori and Ishida 2016) that include both freshwater and marine strains, as well as *Hemiarma marina*. Sequences were aligned using the MAFFT algorithm (Katoh and Standley 2013) and were slightly manually corrected with the help of BioEdit (Hall 1999). The corrected alignment was analyzed with the maximum likelihood (ML) method using RAxML v.8.2.12 (Stamatakis 2014) applying the (GTR) model + Γ + I model of rate heterogeneity with 1000 replicates. Pairwise distances were calculated with BioEdit (Hall 1999). Bayesian analysis was carried out with MrBayes on the XSEDE (3.2.7a) tool (Huelsenbeck and Ronquist 2001; Ronquist et al. 2012) using the (GTR) model + Γ + I with a sampling frequency for the Markov chain set to 10. The stop value of topological convergence diagnostic was set to 0.01. The number of generations/cycles for the MCMC algorithm was set initially to 1,000,000 and was reached after 256,000 generations. Unalignable sites were treated as missing data. The final version of the tree was edited using MEGA X (Kumar et al. 2018), FigTree (Version 1.4.4, https://github.com/rambaut/figtree/releases), and Inkscape (Version 1.0.1) (https://www.inkscape.org/). P‐distances were calculated by aligning two sequences, and after cutting the unaligned front and back sequence sites, they were aligned again, and the individual distance was calculated in percent.

### 2.4 Synapomorphy Analysis

To identify synapomorphic sequence motifs that were shared within the phylogenetic clades, we used a custom Python script built on the Biopython library (Cock et al. 2009) to screen the alignment. The script detected unique, non‐overlapping motifs in the conserved regions of the alignment that occurred in less than 10% of sequences outside the clade. The detected motifs were carefully checked manually to avoid redundancy. Similarities as well as differences were visualized using Inkscape (Version 1.0.1) (https://www.inkscape.org/). Differences in nucleotides within the clades were shown using the IUPAC letter code.

## 3 Results

### 3.1 Morphology

**FIGURE 1:**
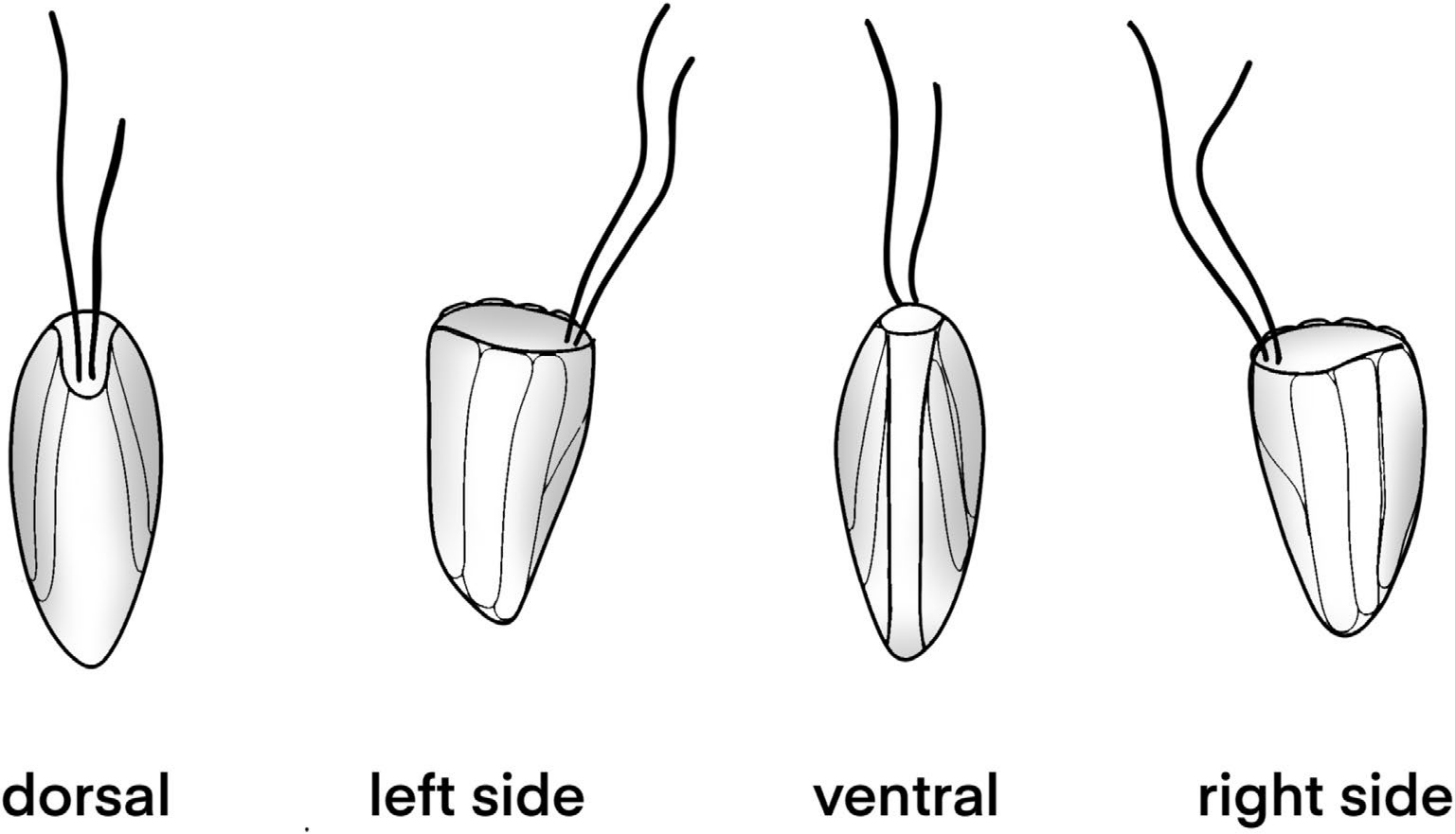
Illustration of cell orientation for goniomonads following the terminology of Mignot (1965).

All “*Goniomonas*‐like” strains investigated in this study shared the following morphological characteristics: an anteriorly truncated cell body, rounded at the posterior end, a cell covered with periplast plates, two flagella arising laterally at the cell anterior, and a band of ejectosomes appearing transversely at the anterior end of the cell. Those basic characteristics varied especially between the freshwater and marine clades, with freshwater strains generally being larger in cell size and more elongated in shape, whereas marine strains were overall smaller and more roundish (Table 1). A contractile vacuole is generally located on the ventral side below the band of ejectosomes in the anterior part of cells, clearly visible in freshwater strains but less visible in marine strains. For determination of the ventral and dorsal sides of the cell, we decided to follow the terminology of Mignot (1965) that designates the narrow side, where the flagella emerge, as the dorsal surface and the opposite narrow side as the ventral surface. The two wide sides are determined as left and right sides (Figure 1).

**TABLE 1:** Landscape table of morphological characteristics summarized for new species and strains investigated in this study.

Species	CL [μm]	CW [μm]	AF [μm]	PF [μm]	Cell shape	Flagella		Periplast plates	Ejectosomes	Origin	18 rDNA
	Mean ± SD	Mean ± SD	Mean ± SD	Mean ± SD		Characteristics	Orientation		Mean ± SD		Reference
*Goniomonas truncata* Stein 1878, emend. (HFCC235)	7.3 ± 1.0 μm, *n* = 22	5.5 ± 0.6 μm, *n* = 22	5.4 ± 0.6 μm, *n* = 17	4.9 ± 0.5 μm, *n* = 16	Slightly elongated, ovoid to pear‐shaped cell body, rounded towards posterior end, tapered, angularly truncated anterior end	Both flagella are equipped with fine hairs, the shorter flagellum carries additional spikey appendages	Both flagella are directed anterior‐laterally	Five irregularly shaped periplast plates on the left and six on the right side, *n* = 14	Average 2.3 ± 0.4 μm, *n* = 7	Freshwater	PX132651
*Goniomonas rhenensis* n. sp. (HFCC251)	9.3 ± 0.4 μm, *n* = 20	6.4 ± 0.8 μm, *n* = 20	5.8 ± 0.7 μm, *n* = 20	4.6 ± 0.5 μm, *n* = 20	Slightly elongated, ovoid to pear‐shaped cell body, rounded towards posterior end, tapered, angularly truncated anterior end	Both flagella are smooth without visible appendages	Both flagella are directed anterior‐laterally	Six irregularly shaped periplast plates on the right side and four on the left side, *n* = 7	1.7 ± 0.1 μm, *n* = 6	Freshwater	PX132652
*Aquagoniomonas mylnikovii* n. sp. (HFCC23)	7.7 ± 1.1 μm, *n* = 19	4.7 ± 0.5 μm, *n* = 20	5.5 ± 0.8 μm, *n* = 8	4.0 ± 1.2 μm, *n* = 8	Elongated, lean cell body, rounded, slightly tapered towards posterior end, angularly truncated anterior end	Both flagella are smooth without visible appendages	Both flagella are directed anterior‐laterally	Four irregularly shaped periplast plates on the left side and five on the right side, *n* = 9	2.0 ± 0.2 μm, *n* = 8	Freshwater	PX132649
*Aquagoniomonas atacamiensis* n. sp. (HFCC5007)	7.7 ± 0.8 μm; *n* = 15	5.0 ± 0.6 μm, *n* = 15	7.6 ± 0.1 μm, *n* = 3	5.6 ± 0 μm, *n* = 3	Slightly elongated ovoid cell body, rounded towards posterior end, angularly truncated anterior end	Both flagella are smooth without visible appendages	Both flagella are directed anterior‐laterally	Four irregularly shaped periplast plates on the left side and five on the right side, *n* = 8	2.0 ± 0.5 μm, *n* = 4	Freshwater	PX132654
*Limnogoniomonas fijiensis* n. sp. (HFCC162)	9.3 ± 1.1 μm, *n* = 20	9.3 ± 1.1 μm, *n* = 20	6.8 ± 1.1 μm, *n* = 14	5.8 ± 0.5 μm, *n* = 13	Elongated cell body, rounded towards posterior end, angularly truncated and obtuse anterior end	Both flagella are smooth without visible appendages	Both flagella are directed anterior‐laterally	Four irregularly shaped periplast plates on the left side and five on the right side, *n* = 9	2.2 ± 0.4 μm, *n* = 7	Freshwater	PX132650
*Baltigoniomonas juergensii* n. sp. (HFCC22)	7.7 ± 0.9 μm, *n* = 20	6.1 ± 0.6 μm, *n* = 20	6.7 ± 0.6 μm, *n* = 20	6.1 ± 0.6 μm, *n* = 20	Ovoid cells with a truncated anterior end	Anterior flagellum equipped with hairs, posterior flagellum is smooth	Anterior flagellum is directed anterior‐laterally, posterior flagellum is directed towards the cell body and oftentimes carried laying over the cell	Four rectangular periplast plates on the left side and three on the right side, *n* = 5	Not observed	Brackish	PX132648
*Marigoniomonas ulleungensis* n. comb. (UH_01; MPRBK‐0001)	2–6 μm	3–5 μm	NA	NA	NA	Two almost equal flagella, slightly longer than cell length, anterior flagellum with hairs, other, laterally oriented flagellum is smooth	One flagellum orientated laterally	Three on the left side and four on the right side	NA	Marine	PQ436815, PQ432910
*Marigoniomonas lingua* n. comb. (GW; MPRBK‐0002)	3–6 μm	3–5 μm	NA	NA	NA	Two almost equal flagella, slightly longer than cell length, anterior flagellum bears hairs whereas the posterior one is smooth	NA	Three on the left side and five on the right side	NA	Marine	PQ448234, PQ448237
*Marigoniomonas* sp. (HFCC841)	4.4 ± 0.4 μm, *n* = 20	3.7 ± 0.5 μm, *n* = 20	4.9 ± 0.6 μm, *n* = 21	6.77 ± 1.12 μm, *n* = 17	Roundish, slightly d‐shaped cells with a truncated anterior end	Anterior flagellum covered with hairs, posterior flagellum is smooth	Anterior flagellum directed anterior‐laterally, posterior flagellum is directed towards the cell body and is often carried laying over the cell	Five rectangular periplast plates on the left side and four on the right side, *n* = 8	1.7 ± 0.1 μm, *n* = 15	Marine	PX232375
*Marigoniomonas* sp. (HFCC272)	4.2 ± 0.5 μm, *n* = 19	3.8 ± 0.5 μm, *n* = 20	5.0 ± 0.5, *n* = 17	4.0 ± 0.5 μm, *n* = 18	Almost round, slightly pointed towards posterior end, truncated anterior end	Both comparably thick, smooth and short, anterior flagellum is slightly longer	Anterior flagellum directed anterior‐laterally, posterior flagellum is directed towards the cell body	No data due to methological difficulties	1.7 ± 0.2 μm, *n* = 16	Marine	PX222343
*Thalassogoniomonas amphinema* n. comb. (HFCC1666)	4.2 ± 0.5 μm, *n* = 19	3.8 ± 0.5 μm, *n* = 19	5.1 ± 0.7 μm, *n* = 16	7.4 ± 0.8 μm, *n* = 18	Cup shaped, slightly elongated, with tapered posterior and truncated, obtuse anterior end	Two smooth flagella of unequal length	Anterior flagellum directed anterior‐laterally, posterior flagellum generally directed towards the cell body	No data due to methological difficulties	1.9 ± 0.2 μm, *n* = 12	Marine	PX132653
*Poseidogoniomonas* sp. (HFCC157)	4.7 ± 0.5 μm, *n* = 18	3.5 ± 0.5 μm, *n* = 18	5.0 ± 0.8 μm, *n* = 15	6.8 ± 1.1 μm, *n* = 14	Roundish, slightly elongated cell body, rounded towards posterior end, truncated anterior end, sides almost parallel to each other	Anterior flagellum carries several fine hairs, posterior flagellum smooth	Anterior flagellum is directed anterior‐laterally, posterior flagellum is directed towards the cell body often curved over the cell body	Cells carry five rectangular periplast plates on the right side and four on the left side, *n* = 12	1.6 ± 0.1 μm, *n* = 5	Marine	PX222342
*Poseidogoniomonas duplex* n. comb. (PT_04; MPRBK_0003)	4–6 μm	3–4 μm	NA	NA	NA	Shorter anterior flagellum slightly longer than cell lenght, bears hairs, posterior flagellum is smooth and 1.5 times longer than cell length	NA	Five on the left side and three on the right side	NA	Marine	PQ436667, PQ436816
*Cosmogoniomonas martincerecadai* n. comb. (CCAP980/2)	5.6 ± 0.51 μm, *n* = 90	3.5 ± 0.57 μm, *n* = 90	5.18 ± 0.44 μm, *n* = 45	6.32 ± 0.45 μm, *n* = 45	Anterior truncate oval shape	Two flagella of unequal length, appendages visible only on the short flagellum	Two flagella, one the long one, which trails behind, one shorter one, held straight while swimming	No data	No data	Marine	EU047707
*Neptunogoniomonas avonlea* n. comb. (PLY722)	8–11 μm				Flattened cell body, somewhat truncated at the anterior	Subequal, slightly longer than the cell, fine hairs on anterior flagellum, smooth posterior flagellum	One flagellum is directed forward, one runs backward towards the cell posterior	Four dorsal, 3 ventral (left/right)		Marine	JQ434475, JQ434476

Abbreviations: AF, anterior flagellum; CL, cell length; CW, cell width; PF, posterior flagellum.

**FIGURE 2:**
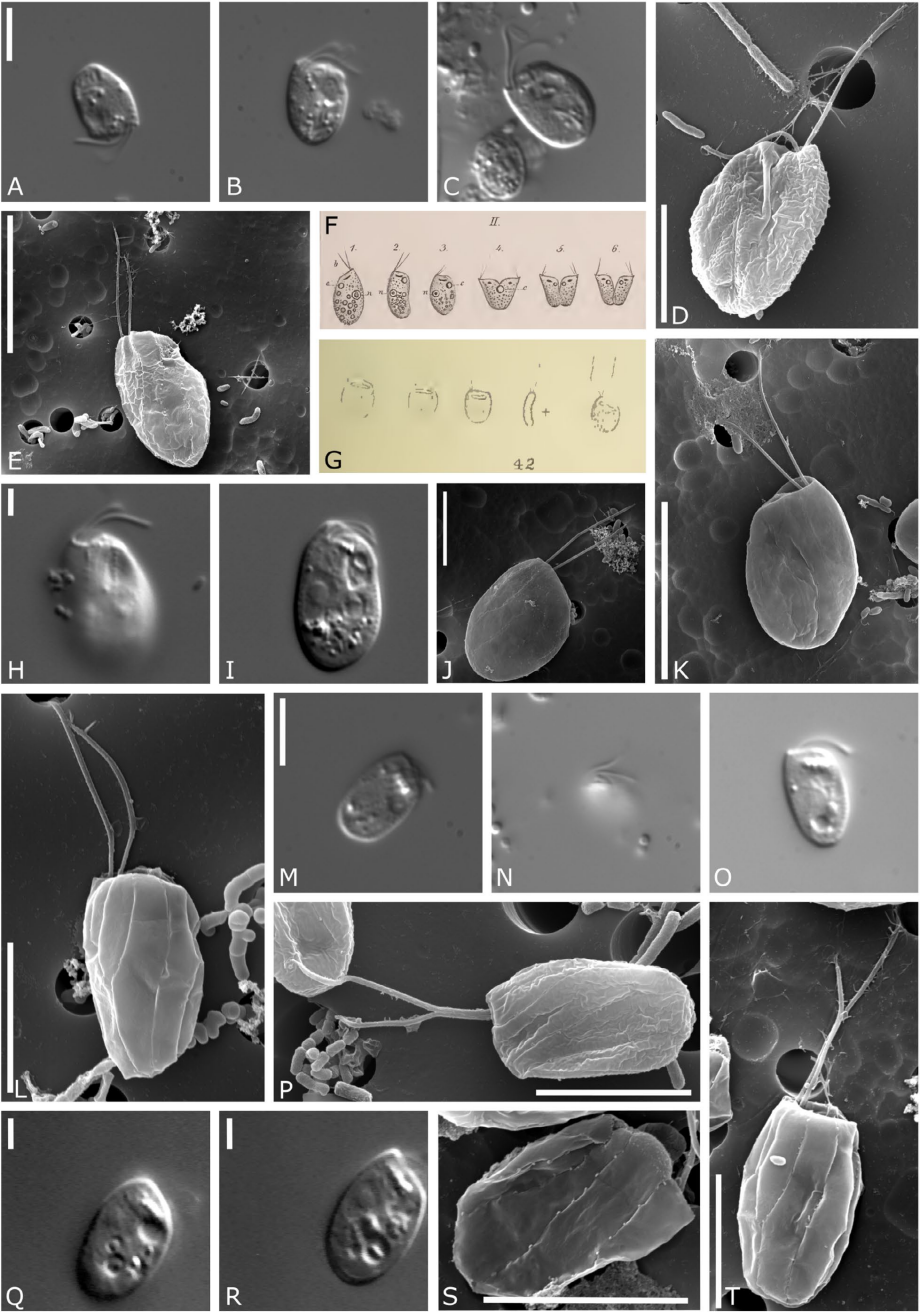
Light and electron micrographs and drawings of the genera *Goniomonas* and *Aquagoniomonas* n. g. et n. sp. (A–E) *Goniomonas truncata* (bar indicates: A, D – 5 μm, E – 10 μm), (F) drawing of *Goniomonas truncata* (from Stein 1878), (G) drawing of *Monas truncata* (from Fresenius 1858), (H–K) *Goniomonas rhenensis* n. sp. (bar indicates: H – I 2 μm, J – 5 μm, K – 10 μm), (L–P) *Aquagoniomonas mylnikovii* n. g et. n. sp. (bar indicates: L, M, P – 5 μm), (Q–T) *Aquagoniomonas atacamiensis* n. g. et n. sp. (bar indicates: Q, R – 2 μm, S, T – 5 μm).

**FIGURE 3:**
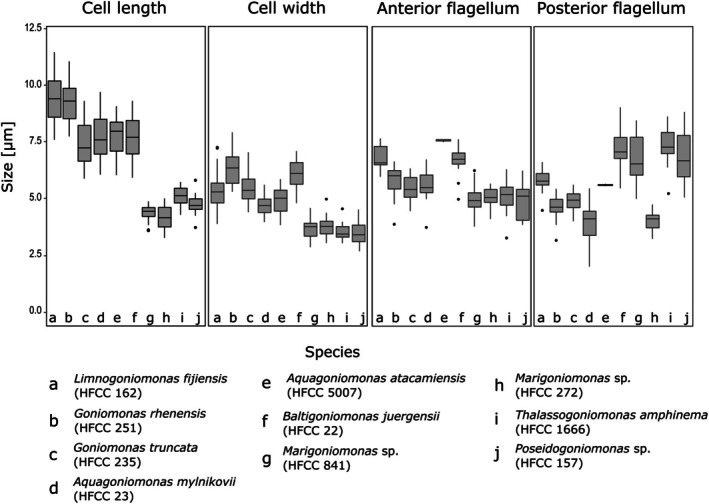
Boxplots showing the comparison of morphological measurements of the described goniomonads. Letters in the box plots indicate the different species. (a) *Limnogoniomonas fijiensis* n. g. et n. sp.; (b) *Goniomonas rhenensis* n. sp.; (c) *Goniomonas truncata*, (d) *Aquagoniomonas mylnikovii* n. g. et n. sp., (e) *Aquagoniomonas atacamiensis* n. g. et n. sp.; (f) *Baltigoniomonas juergensii* n. g. et n. sp.; (g) *Marigoniomonas* sp. (HFCC841); (h) *Marigoniomonas* sp. (HFCC272); (i) *Thalassogoniomonas amphinema* n. comb.; (j) *Poseidogoniomonas* sp. (HFCC157).

**FIGURE 4:**
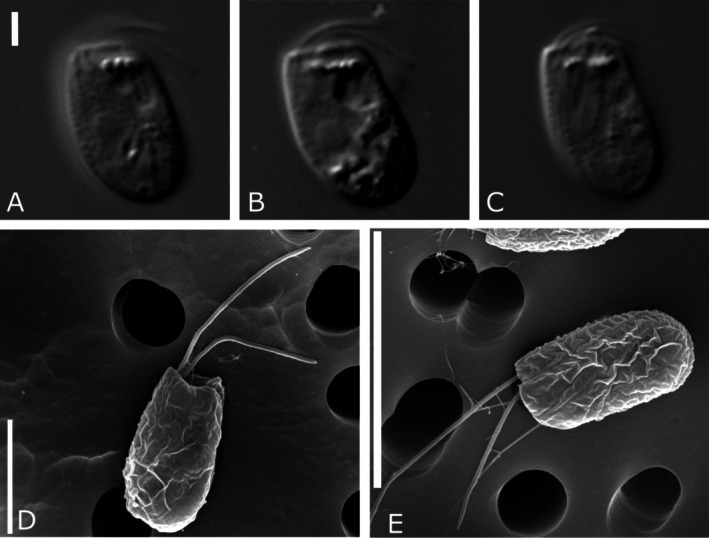
Light and electron micrographs showing *Limnogoniomonas fijiensis* n. g. et. n. sp. (A–E; bar indicates: A – 2 μm, D – 5 μm, E – 10 μm).

**FIGURE 5:**
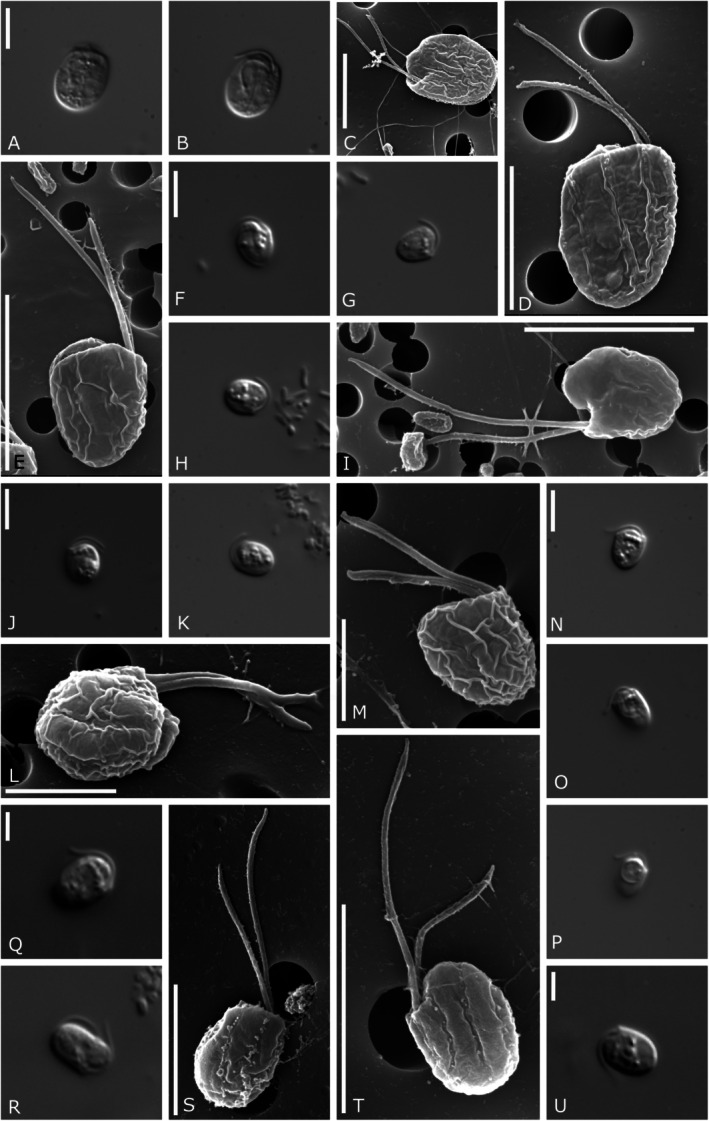
Light and electron micrographs showing: (A–D) *Baltigoniomonas juergensii* n. g. et n. sp. (bar indicates A, D – 5 μm), (E–I) *Marigoniomonas* sp.—HFCC841 (bar indicates: E, F, I – 5 μm), (J–M) *Marigoniomonas* sp.—HFCC272 (bar indicates: J – 5 μm, L, M – 2 μm), (N–P) *Thalassogoniomonas amphinema* n. comb. (bar indicates: N – 5 μm), (Q–U) *Poseidogoniomonas* sp. (HFCC157, bar indicates: Q, U – 2 μm, S – 4 μm, T – 5 μm).

Two out of 10 analyzed strains for this study (HFCC235 and HFCC251) could be assigned to the genus *Goniomonas* (Figure 2, A–G resp. H–K) isolated from freshwater. The cell shape was always laterally compressed, slightly elongated ovoid, and sometimes pear‐shaped (HFCC251). The elongated shape makes the left and right margins of the cells appear almost parallel. Two other goniomonad strains (HFCC23 and HFCC5007) were assigned to the newly designed freshwater clade *Aquagoniomonas* n. g. (Figure 2, L–P resp. Q–T, Figure 3). Morphologically similar, though genetically distinct, were the following clades: Strain HFCC162 (Figure 4) was assigned to the new genus *Limnogoniomonas* n. g. Morphological observations for strain HFCC22 assigned to the genus *Baltigoniomonas* n. g. (Figure 5A–D) show a similar cell size as *Limnogoniomonas* n. g. Both strains are free‐living and display a similar swimming behavior as *Goniomonas* and *Aquagoniomonas* n. g. Cells are laterally compressed and slightly elongated. They carry two flagella of unequal length, of which one possesses stiff flagellar hairs. Strains HFCC841 and HFCC272 both fall into the newly created genus *Marigoniomonas* n. g. (Figure 5, E–I and J–M). In relation to the cell size, the flagella are much longer compared to the freshwater strains. Both strains show a roundish, rather compact cell shape, not elongated in contrast to the freshwater genera. Cells of strain HFCC 841, which were isolated from the Chilean Pacific coast near Antofagasta, were 3.6–4.9 μm (average 4.4 ± 0.4 μm, *n* = 20) long and 2.9–4.6 μm (average 3.7 ± 0.5 μm, *n* = 20) wide. Flagella were of unequal length, with the anterior flagellum being approx. 1/3 shorter, with 4.2–6.3 μm (average 4.9 ± 0.6 μm, *n* = 21) length and covered with hairs, while the posterior flagellum was smooth and directed towards the cell body and is often carried lying over the cell, 5.1–8.5 μm (average 6.77 ± 1.12 μm, *n* = 17) long. Cells carry five rectangular periplast plates on the left side and four on the right side. Periplast plates could not be analyzed for strain HFCC272 due to difficulties with the preparation. Cells of strain HFCC272, which were isolated from the Atlantic coast of Madeira from 950 m depth off Ribeira, were 3.3–5.0 μm (average 4.2 ± 0.5 μm, *n* = 19) long and 3.1–5.0 μm (average 3.8 ± 0.5 μm, *n* = 20) wide. The anterior flagellum was slightly longer and directed anterior‐laterally, with a 5.0 ± 0.5 μm (4.1–5.7 μm, *n* = 17) length; the posterior flagellum was directed towards the cell body and was 3.2–4.8 μm (4.0 ± 0.5 μm, *n* = 18) long. In addition to the prominent band of ejectosomes, a smaller nodule‐like ejectosome between the periplast plates was visible.

Another genetically distinct strain, HFCC1666, was placed into the newly described genus *Thalassogoniomonas* n. g. (Figure 5, N–P). Cells were obovate to cup‐shaped, with two unequally long flagella, the posterior carried over the cell body. The genus *Poseidogoniomonas* so far comprises two new isolates: one from our own study (HFCC157, designated *Poseidogoniomonas* sp., Figure 5, Q–U) and *P. duplex* n. comb. (Phanprasert et al. 2025 emend.). Both strains have cells of about 4–6 μm length and 3–4 μm width and carry two flagella, of which the shorter anterior flagellum carries fine hairs and the longer posterior flagellum is smooth. Strains differ in the number of periplast plates.

### 3.2 Phylogenetic Analysis

**FIGURE 6:**
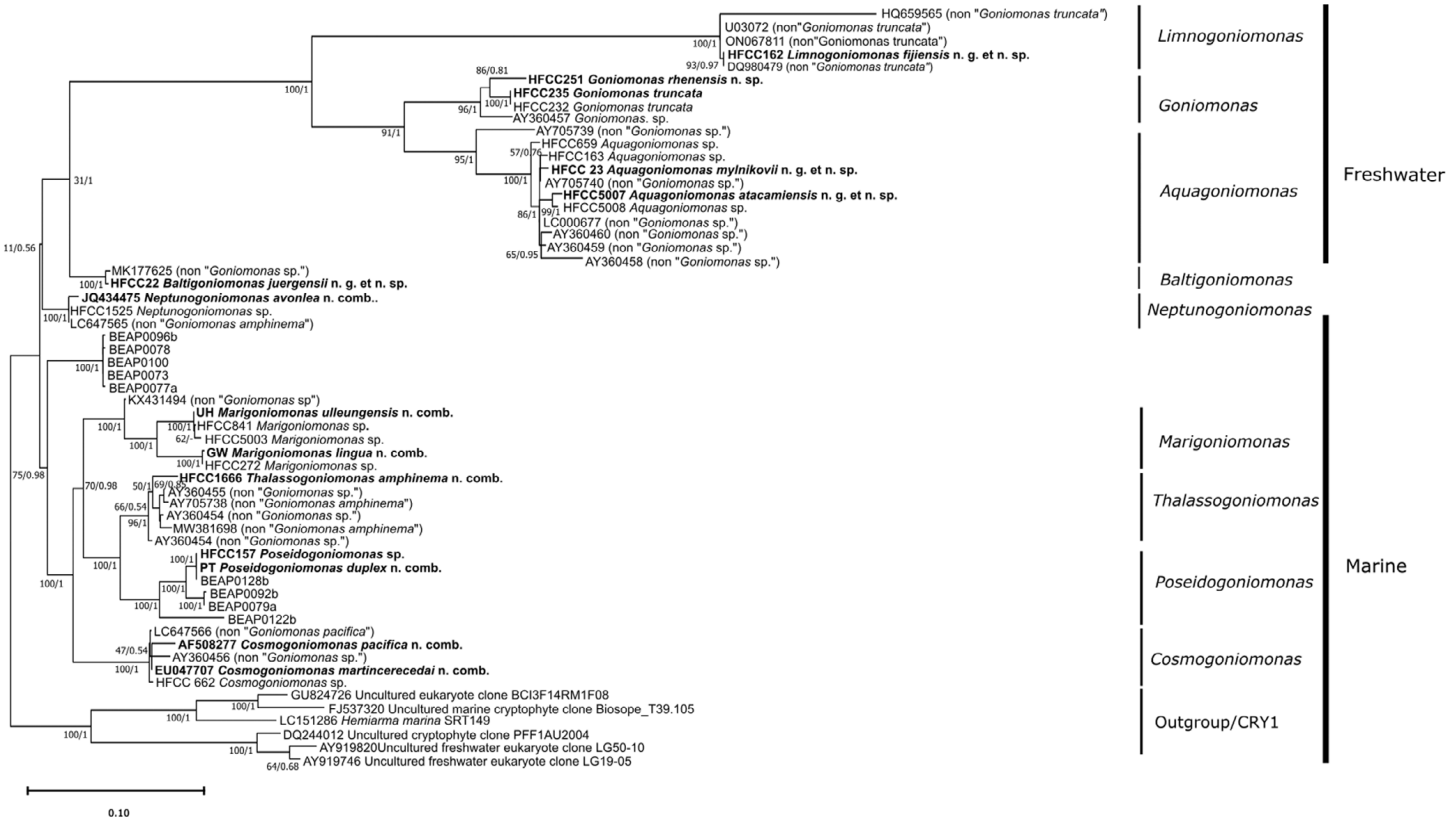
Maximum likelihood (ML) phylogenetic tree based on sequences of 18S rDNA. Investigated strains are marked in bold. Numbers indicate bootstrap values of ML analysis as well as Bayesian analysis for the branches, “‐” indicates a differing topology for Bayesian analysis. The unnamed sequence cluster containing sequences with the label BEAP will be discussed in further studies. Within this cluster all sequences belong to strains isolated from the Mediterranean Ocean.

The analysis of the partial 18S rDNA data shows a clear separation between the freshwater and marine clades. The freshwater cluster is formed by the genera *Goniomonas* and the newly described genus *Limnogoniomonas* n. g. and *Aquagoniomonas* n. g. The marine cluster is formed by *Marigoniomonas* n. g., *Thalassogoniomonas* n. g., *Poseidogoniomonas* n. g., and *Cosmogoniomonas* n. g., and there is one genus, up to now only recorded from brackish water, *Baltigoniomonas* n. g. (Figure 6), closely related to *Neptunogoniomonas* n. g.

Among the newly isolated and analyzed strains, two cluster within the freshwater clade (HFCC251, HFCC235) of *Goniomonas*, one in the freshwater clade of *Limnogoniomonas* n. g. (HFCC162), and two in the freshwater clade of *Aquagoniomonas* n. g. (HFCC23 and HFCC5007). Among the marine isolates, one clusters in the clade *Baltigoniomonas* n. g. (HFCC22), two in the clade *Marigoniomonas* n. g. (HFCC 841 and HFCC272) with two from the study of Phanprasert et al. (2025), one in the clade *Thalassogoniomonas* n. g. (HFCC1666), and one in the clade *Poseidogoniomonas* n. g. (HFCC157). In many cases, Bayesian analysis produced stronger support of the clades than ML analysis; weakly supported clades can be found both in freshwater and marine groups.

Among the freshwater clades, the neotype of *G. truncata* clusters together with two other sequences isolated from the River Rhine. A pairwise comparison revealed a 0.0% difference to strain HFCC232 and a 3.8% difference to strain HFCC251, indicating the existence of two distinct species in this cluster, strongly supported by bootstrap values (Table S2). Sequences designated as *Goniomonas* share at least eight unique motifs within conserved regions of the 18S rDNA, with overall only two nucleotide differences in the first and fourth motifs within the clade. Both differences occur in sequence AY360457. Sequence motifs in the *Goniomonas* clade share most characters with the *Aquagoniomonas* clade, followed by *Limnogoniomonas*. More dissimilarities were found in comparison to the marine and brackish water clades.

The large clade of the newly described genus *Aquagoniomonas* n. g. houses eleven sequences from strains originating from different freshwater environments. The newly described species, *Aquagoniomonas mylnikovii* n. g. et n. sp., isolated from a pond in Borok, Russia, is closely related, though not identical, to a sequence from GenBank (LC000677), and it clusters closely together with another GenBank sequence from a UK freshwater habitat (AY705740) and a strain from our own collection (HFCC163) isolated from the river Rhine with a relatively small genetic distance (Table S2). The clade consists of several undescribed and undetermined strains from various geographic origins (originally assigned to *Goniomonas* despite their large genetic distances). Within this clade, two strains from our own collection appear, both originating from the Rio Loa in the Atacama Desert in Chile. Strain HFCC5007 is closely related to strain 5008. Within this clade itself, genetic differences can be high, up to 9.7% (Table S2). Basal branching of *Aquagoniomonas* as a sister clade to *Goniomonas* is well supported by bootstrap values (> 90/0.9), while support within the large clade is partly weaker. Future studies might further resolve the phylogenetic relationship of *Aquagoniomonas* (Figure 6).

In the clade *Limnogoniomonas* n. g., strain HFCC162, isolated from a pond on the island of Fiji, clusters closely together with two sequences formerly designated as *G. truncata* (Table S2). The genus *Limnogoniomonas* clusters as a sister clade to the other freshwater taxa and is strongly supported by bootstrap values (> 90/0.9), and there are no dissimilarities within the chosen motifs within this clade. While it shares some similarities with the other freshwater clades, it lacks a number of longer inserts present in the others that lead to the individual branch. The sequence HQ659565 displays a longer branch characterized by a minor number of shorter inserts that the other *Limnogoniomonas* n. g. do not possess (Figure 6).

**FIGURE 7:**
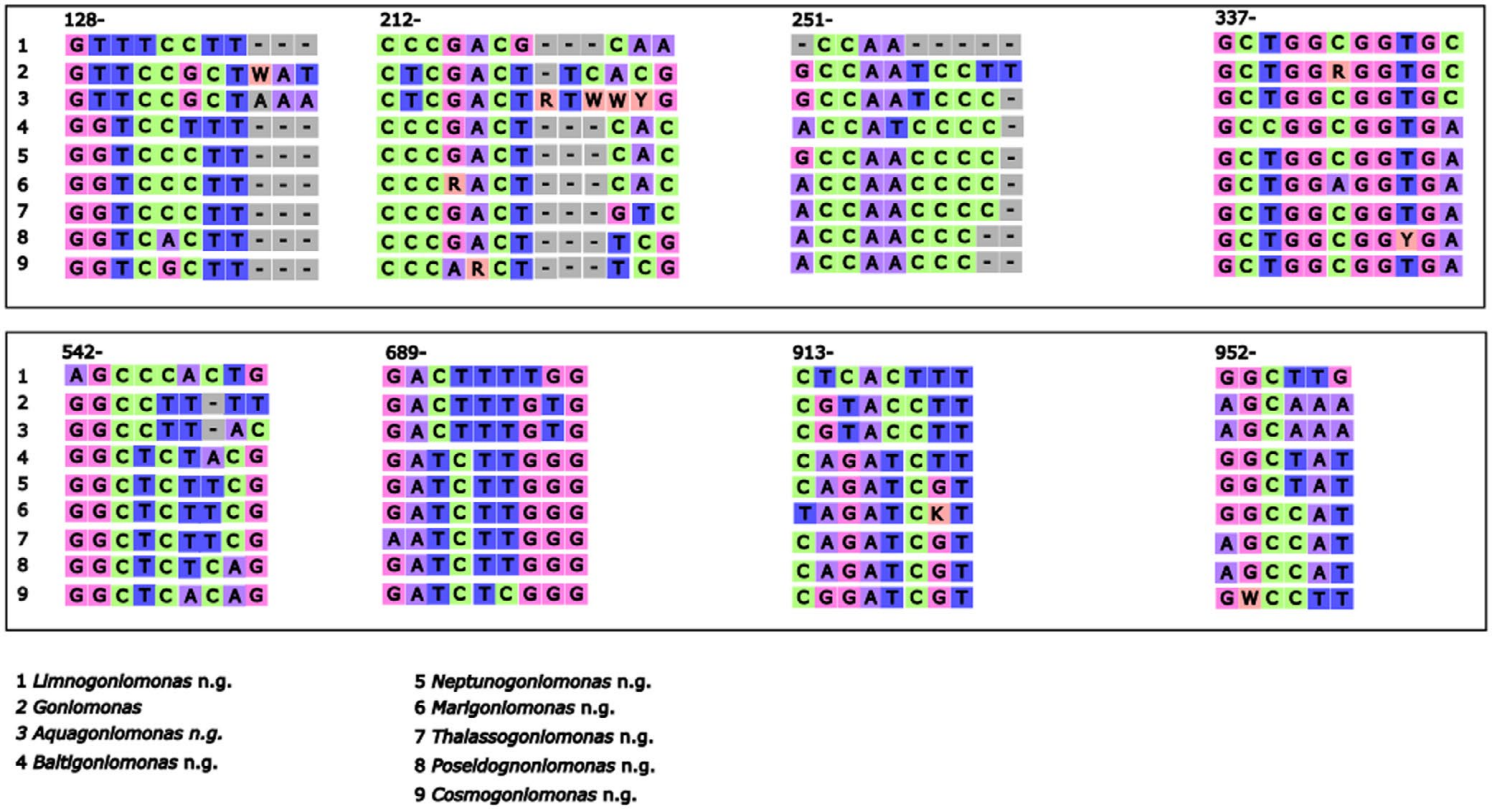
Diagram illustrating synapomorphic motifs within the 18S rDNA across nine clades, highlighting dissimilarities between the sequence clusters. The numbers above each motif represent the start position of the motif within the original alignment of this study (Supporting Information). Variations in the motifs underscore evolutionary relationships and distinctions.

The marine species clustered more separately from each other, with a shorter branch than isolates from freshwater habitats. Strain HFCC22 from brackish environment (Baltic Sea) clusters together with a GenBank sequence (MK177625) originating from a freshwater pond on an island in Korea with only a relatively small genetic difference (Table S4). The branch is well supported by bootstrap values, and the chosen synapomorphy motifs were completely consistent between the two sequences (Figure 7). The clade *Baltigoniomonas* n. g., with *B. juergensii* n. g. et n. sp., originating from brackish waters, is separated from *Neptunogoniomonas* n. g. due to several single nucleotide differences in the synapomorphy motifs (Figure 7). *Neptunogoniomonas* n. g. includes the most recently described “*G*.” *avonlea* (now *Neptunogoniomonas avonlea* n. comb.) isolated from Canadian marine waters and two other sequences, one originating from our own collection (HFCC1525, isolated by Sabine Schiwitza from Danish coastal waters) and one sequence that had originally been assigned to “*G. amphinema*” LC647565. On the level of SSU rDNA, HFCC1525 has differences from “*G*.” *avonlea* (JQ434475; Table S5), but is identical in nuclear LSU rDNA (not included in this study). We also added several sequences (BEAP—**B**iology and **E**cology of **A**bundant **P**rotists Lab, Institute of Evolutionary Biology, CSIC‐Universitat Pompeu Fabra, Barcelona) originating from organisms sampled in the Mediterranean Ocean kindly provided by Daniel Richter and Daryna Zavadska and their group to our phylogenetic analysis. Of those sequences, five formed an individual cluster supported with high bootstrap support. These potentially new species and a potentially new genus have to be described in future studies when morphological data are available.

The remaining marine clades overlap regarding their synapomorphic motifs, though each clade has its separate identity (Figure 7). The large morphological and genetic variety of the additional marine species is structured into four clades.

Another clade with strong bootstrap support belongs to the newly created genus *Marigoniomonas* n. g. It comprises the closely related strains HFCC841 and HFCC5003 (Table S7). Both originated from the Pacific at the coast of the Atacama Desert in Chile and are highly similar on the basis of the SSU rDNA. The strain HFCC272 from the North Atlantic clusters beside the two sequences from the Pacific with a distance to strain HFCC841. The first motif is not present in HFCC272 and HFCC5003 due to the length of the sequences (Figure 7). In two other motifs (two and seven), it is HFCC272 that shows a total of two single nucleotide differences underlining its separate position in the cluster. The two species *Marigoniomonas ulleungensis* n. comb. and *Marigoniomonas lingua* n. comb. recently described by Phanprasert et al. (2025) fall into this cluster and were transferred to this new genus.

Six other sequences from marine sites form the clade designated *Thalassogoniomonas* n. g. (Table S7). *Poseidogoniomonas* n. g. is a marine clade comprising six sequences, of which one belongs to our own collection, one to the recent study of Phanprasert et al. (2025), and the other four originate from the Mediterranean Ocean (Richter and Zavadska, pers. communic., see above). The newly emended strain *P. duplex* n. comb. (formerly *Goniomonas duplex*, Phanprasert et al. 2025) serves as a type species for this clade. The genetic diversity in the clade is relatively large (Table S7); still, this part of the tree is robust based on bootstrap values, and we could only detect one nucleotide difference between the sequences within the chosen motifs. In motif four, strains HFCC157, *P. duplex* n. comb., and BEAP128b both display a thymine (T) at position 345, where the other two have a cytosine (C). The strain HFCC 1666 was designated as the type species of this clade and will serve as a neotype for “*Goniomonas” amphinema*.

A potentially basal genus is a clade consisting of sequences originally named either “*Goniomonas* (aff.) *amphinema*” or “*Goniomonas*” *pacifica* (*Cosmogoniomonas martincerecedai* n. comb., resp. *C. pacifica* ), yet none of the sequences could serve as a type for those species. The clade is enlarged by a strain originating from our own collection, HFCC662, isolated from the Maldives, with only minor genetic distances on the SSU rDNA level to strain LC674566 that was originally assigned to “*G*.” *pacifica*. This sequence is also very close to the sequence defined as “*G*. aff. *amphinema*” (EU047707), with only two nucleotides being different (Table S6).

The sister group relationship of *Thalassogoniomonas, Poseidogoniomonas, Cosmogoniomonas*, and *Marigoniomonas* is robust and supported by the chosen synapomorphy motifs (Figure 7).

## 4 Discussion

### 4.1 Divergence of Freshwater and Marine Strains and New Genera

During this study, we investigated ten different cryptophyte strains with a *Goniomonas*‐like morphology. Apart from designating a valid neotype for *G. truncata* , we split the taxonomically not well‐resolved genus into three freshwater and six marine/brackish water genera and described six new species based on morphological and/or phylogenetic findings.

A possible splitting of the genus *Goniomonas* into further clades has already been proposed in earlier studies (Kim and Archibald 2013; Von Der Heyden et al. 2004). Von Der Heyden et al. (2004) compared the branching depth between freshwater and marine species to be similar to what separates most of the prymnesiophyte genera. The phylogenetic results and the robust values at the basal split between those two groups support the demand to especially separate those two groups. We found the largest genetic difference (38% on the basis of the 18S rDNA) between the newly defined neotype of *G. truncata* (strain HFCC 235) and *Poseidogoniomonas* sp. (strain HFCC 157), a strain from one newly described marine genus. But even between an established species like the formerly known “*G*. (aff.) *amphinema*” (EU047707, now *Cosmogoniomonas amphinema* n. comb.) and *G. truncata* we found a genetic difference of 21%. The formerly known “*G. amphinema*,” “ *G. pacifica*,” and “*G. avonlea*” were therefore sorted to different genera, *Thalassogoniomonas* n. g., *Cosmogoniomonas* n. g. resp. *Neptunogoniomonas* n. g. Earlier studies focusing on the 18S rDNA phylogeny of goniomonads (Deane et al. 2002; Von Der Heyden et al. 2004) showed that the major difference between the freshwater and marine clades is based on several inserts that some freshwater species possess and that are absent in marine goniomonads. This pattern is confirmed by our newly described species. Deane et al. (2002) and Von Der Heyden et al. (2004) also found that one sequence (UO3072) from freshwater is lacking the insert typical for all other freshwater goniomonads. The newly described genus *Limnogoniomonas* n. g. includes this specific sequence. Additionally, all other sequences that appear in the *Limnogoniomonas* clade are lacking this insert, resulting in an individual branch, apart from the other freshwater genera. This is also reflected by the performed synapomorphy analysis. In the first motif, only *Goniomonas* and *Aquagoniomonas* show the full motif, whereas all other clusters, including *Limnogoniomonas*, display a gap after position 135. The well‐supported branching (as well as the large genetic distance) of the type sequence HFCC162 to the new neotype of *G. truncata* (HFCC235) may validate the erection of a new genus, *Limnogoniomonas* n. g. The clade does also house a sequence (ON067811) originating from a study focusing on cryptic and aplastic cryptophytes (Simek et al. 2023). The study designates, but does not scientifically describes the strain as *Pseudogoniomonas* and sorts it as a sister clade to the aplastidic CRY1 lineage (Shiratori and Ishida 2016; Shalchian‐Tabrizi et al. 2008) and *Goniomonas*. However, the sequence was deposited in GenBank not under the name *Pseudogoniomonas* but *G. truncata*. Although this is initially misleading, it ultimately corresponds to our argument above, since the study separates *Pseudogoniomonas* from *Goniomonas* as we do here for *Limnogoniomonas* n. g.

Apart from the divergence between freshwater and marine/brackish strains, there was also a remarkable genetic diversity even within the different genera. In our study, the *Goniomonas* freshwater clade houses four sequences, including one of a newly described species. Between the neotype and *Goniomonas rhenensis* n. sp., we calculated a pairwise distance on the basis of the 18S rDNA of approx. 3.8%. For species in the genus *Aquagoniomonas* n. g., a pairwise distance of 6% (between sequences of HFCC659 and AY360458) and up to 9.67% (between sequences AY705739 and AY360458) was recorded. Within the marine genera, we found the following maximum genetic differences with regard to the 18S rDNA gene: *Baltigoniomonas* n. g. 0.31%, *Neptunogoniomonas* n. g. 0.41%, *Marigoniomonas* n. g. 3.4%, *Thalassogoniomonas* n. g. 1.76%, and *Poseidogoniomonas* n. g. 3.4% (Tables S4–S7).

Some flagellate isolates originating from rather remote regions (e.g., *Thalassogoniomonas amphinema* n. comb. from Helgoland) form an individual branch. This may be a sign of allopatric speciation in remote areas/islands (Hernandez‐Hernandez et al. 2021), as it was postulated for percolomonads and other protist species (Arndt et al. 2020; Hohlfeld et al. 2022). Yet, genetic differences between different protist genera can widely vary depending on the genus and family. It is therefore difficult to set up boundaries or thresholds up to where a sequence does still belong to a genus or not. We consider our separation reasonable but are aware that further studies may change the picture again. By looking at the synapomorphy analysis, there are several differences in motifs between freshwater and marine clades and also between the different marine clades. The topology of the freshwater clades is overall more robust, which could stem from the larger number of marine sequences (30 vs. 20 in the freshwater clade), reducing support. The broader topology of the tree is partly well supported, which suggests that the tree correctly reflects the evolutionary relationships at higher taxonomic levels and supports our decision to split the genera and to hopefully clarify the systematic position of strains and sequences in the future. Nevertheless, some positions in the tree remain uncertain, as the branching of the *Baltigoniomonas* and *Neptunogoniomonas* clade to each other as well as to the freshwater and marine clusters highlights their position in‐between the two. The lower bootstrap values within some of the clusters could be attributed to the limited resolution of the 18S marker and could be refined with further multigene resolution. Unfortunately, only very few sequences of other marker genes are available for goniomonads at the moment in public databases that could potentially have resolved some uncertainties.

The observed deep sequence divergence stands in contrast to the rather conserved morphological features of the established genera that hardly allow such a clear differentiation at first glance based on their morphology. Our study shows that features like cell size and even position and length of flagella that have been used to distinguish e.g., the former species *Goniomonas pacifica* and *G. amphinema* from each other have lost their discriminative power, as these characters are also found in other, genetically distinct species. The position, direction, and length of flagella as described for “*G*. aff.” *amphinema* (Martin‐Cereceda et al. 2010) can also be found in species like *Baltigoniomonas juergensii* n. g. et n. sp. and *Poseidogoniomonas* sp. (strain HFCC157) that have at least 6% pairwise distance in the 18S rDNA to the former species. Earlier studies have already noted that even without a close link to 18S rDNA data, different genera of Cryptophyta can show surprisingly similar morphologic features (Deane et al. 2002). Hence, it is necessary to lay a stronger focus on molecular data to define species in this phylogenetic group of organisms and to use morphological features as Supporting Information. Still, we can observe morphological similarities within the newly described genera.

### 4.2 Designation of a Neotype for *G. truncata*

In public databases, around 25–30 sequences are available for phylogenetic analyses of the genera investigated in this study (on the basis of the 18S rDNA). After our phylogenetic analyses, four of these sequences fall into the *Goniomonas* clade. Since its first description (Fresenius 1858), the species *G. truncata* has been analyzed in multiple studies (Bernard et al. 2000; Ekelund and Patterson 1997; Hill 1991; Kugrens and Lee 1991; Patterson and Lee 2000; Vørs 1993), but every time without any genetic material to underpin its taxonomic position. Together with other studies (Von Der Heyden et al. 2004), our studies show how genetically diverse the genus actually is. It is therefore hard to judge whether the publications listed above really analyzed *G. truncata* itself or lumped multiple species into one ever‐increasing description. Kim and Archibald (2013) have completed a helpful table showing how many studies actually described each species of the genus.

The designation of strain HFCC235 as the neotype for *G. truncata* was done, as the morphology of this strain resembles the original description and the sampling origin is close to the area where the original studies have been carried out by Fresenius. Stein (1878) placed the species from *Monas truncata* into the new genus *G. truncata*. The original description by Fresenius (1858) lists the following morphological features: cells are found in freshwater, are colorless, oval to roundish, and 6–10 μm in length; cells are laterally compressed. In addition, the anterior end is truncated and carries two flagella that originate on the side and have a length of about the total cell length. Close to the anterior margin lies a band of a narrow structure (today we know that these are ejectosomes), and close to this band is a contractile vacuole. The swimming behavior is described as trembling with a frequent change in direction. The strain HFCC235 fits well to this original description, with a cell length of 5.9–9.3 μm, an ovoid cell shape, and two flagella with a similar length of 4.5–6.3 μm and 4.0–5.6 μm, respectively. Depending on the individual cell, the characters fall within the description given by Fresenius (1858). It also has a band of ejectosomes on the anterior margin of the cell. Additionally, HFCC235 comes from a freshwater environment and was sampled in Germany, which is assumingly the sampling origin of both Fresenius' and Steins' strains. One could argue that Fresenius gave a rather unspecific description of a goniomonad and that, apart from the length of flagella in ratio to the cell size, his descriptions are quite universal for this genus. Nonetheless, we find this strain appropriate due to the reasons mentioned above and also on the basis of the illustrations that both Fresenius (1858) and Stein (1878) provided.

### 4.3 Combining Phylogenetic and Morphological Data

With *Limnogoniomonas* n. g., we named a clade currently consisting of 4 strains with *Limnogoniomonas fijiensis* n. g. et n. sp. as the type species. *L. fijiensis* n. g. et n. sp. differs from *G. truncata* by having a larger cell length of 7.6–11.4 μm but is similar in width, making the cells much more elongated than those of *G. truncata* . In addition, *L. fijiensis* n. g. et n. sp. has longer flagella than all *Goniomonas* species described within this study. In the synapomorphy motifs, the clade of *Limnogoniomonas* n. g. and *Goniomonas* differs by at least one nucleotide per motif. Their relation to each other is robustly supported by the tree topology (Figures 6 and 7).

Within the freshwater clades, three isolates from the Rhine river cluster closely together (HFCC251, HFCC232, and HFCC235). Another one (HFCC163) appears quite far away and is part of the *Aquagoniomonas* n. g. clade. HFCC235 was designated as the neotype for G. truncata, and HFCC251 was described as the new species *G. rhenensis* n. sp. With a pairwise distance of 3.8%, the type species branches closely, though poorly supported. *G. rhenensis* n. sp. displays larger (both in length and width) cells like *G. truncata* , while the anterior and posterior flagella are of similar length. *G. rhenensis* n. sp. carries six irregularly shaped periplast plates on the right side and four on the left side, while *G. truncata* carries five left and six right side periplast plates, respectively. HFCC232 has an identical sequence to HFCC235 and can be considered a re‐isolation of *G. truncata* from the neotype locality. The chosen motifs on the basis of 18S rDNA revealed a robust relationship between the *Goniomonas* species, additionally supported by the occurrence of the above‐mentioned inserts that they share only with strains of *Aquagoniomonas* n. g.

The strain HFCC23, isolated near Borok, Russia, was described as the new species *A. mylnikovii* n. g. et n. sp. It serves as the type species for this new genus. On the basis of the 18S rDNA, it shows a pairwise distance of 0.47% to its closest relatives, the two undescribed sequences LC000677 and AY705460 isolated in Japan and the UK, respectively. In distinction to *Goniomonas*, *A. mylnikovii* n. g. et n. sp. has a similar cell length but is wider and has a longer posterior flagellum. The cell size is smaller than that of *G. rhenensis* n. sp., and it has a shorter anterior and posterior flagellum than the latter. It carries four periplast plates on the left and five on the right side. HFCC 5007 was designated as *Aquagoniomonas atacamiensis* n. g. et n. sp. and has its type locality in the Rio Loa in Chile. This strain has a pairwise distance of 10.4% to *G. truncata* and 0.61% to its closest relative, a strain also originating from the Rio Loa but found in the mouth area of the river. *A. atacamiensis* n. g. et n. sp. is of similar cell size to the type species *A. mylnikovii* n. g. et n. sp. but carries longer flagella. Both species have four periplast plates on the left and five on the right side. The inner topology of the closely related sequences is mostly well supported, and except for motif two, where there is a higher level of dissimilarities between the sequences/strains, the other motifs are completely identical within the *Aquagoniomonas* clade.

The genera *Neptunogoniomonas* n. g. and *Baltigoniomonas* n. g. comprise three sequences from public databases and two sequences from our own collection. Phylogenetically, the genus is subdivided into two branches that we consider to be different genera, as their relationship is only poorly supported in the tree. One branch contains two species from GenBank, including the species formerly known as *G. avonlea*, now *N. avonlea* n. comb. One sequence originates from Denmark just at the transition zone between the North Sea and Baltic (HFCC1525), and one sequence has marine origin (LC647565). The other branch contains strain HFCC22, originating from the Baltic Sea, described here as *B. juergensii* n. g. et n. sp. It branches together with a sequence from a freshwater pond in Korea (MK177625). Thus, the broader branch consists of strains from brackish, marine, and freshwater environments. This is quite unusual, as all other sequences in the lower half of the phylogenetic tree (Figure 6) have a strictly marine background. Aside from this fact, the morphology of *B. juergensii* n. g. et n. sp. slightly differs from that of *N*. a*vonlea* n. comb. With a cell length of 5.9–9.3 μm and a cell width of 4.8–7.1 μm, it is smaller than the latter, but the ratio of flagella length compared to cell length seems to be similar. Both species carry mastigonemes on one flagellum, whereas the other one appears to be smooth. Both species carry four rectangular periplast plates on the left side and three on the right side. The sequences show a pairwise distance of 3.4% to each other (on the basis of 18S rDNA). The synapomorphy analysis revealed several similarities between the two genera, but also enough differences to justify the split. The motifs for the two clusters are completely identical within the clades.

The strains HFCC841 and HFCC5003 form an individual branch together with another sequence from our own collection, HFCC 272, two recently described species from South Korean sandy beaches (Phanprasert et al. 2025), and a more distant sequence from a public database (KX431494). Together they form the new genus *Marigoniomonas* n. g., with *M. ulleungensis* n. comb. designated as the type species. Interestingly, *M*. *ulleungensis* n. comb. branches closely together with our strains HFCC841 and HFCC5003; all strains originated from coastal waters of the Pacific Ocean. On the basis of the compared sequences, our isolate HFCC5003 had only two nucleotide differences; however, at our present stage of knowledge, we cannot decide whether this is a character discriminating different species. The next sequence in the branch, HFCC272 and *M. lingua* n. comb., has a difference of about 3.5%. Our strain HFCC272 is morphologically similar to *M. lingua* n. comb., though not identical regarding the 18S rDNA (≤ 7 nucleotide differences). Due to methodological problems, we were not able to carry out SEM studies of strain HFCC272. At least, the two strains are of similar size. Whether or not these isolates belong to the same species has to be investigated in future studies.

The sister clade to *Marigoniomonas* n. g. houses a variety of sequences from all over the world. Some were designated as “*G. amphinema”*, assumingly on the basis of morphological observations, even though they appear genetically quite distant from the clade where “*G*. aff. *amphinema”* (EU047707) appears. Outside of this clade branches the newly designated HFCC1666, *T. amphinema* n. comb. It is closely related to the sequence AY360454. There are no other described species that could be analyzed for comparison within this clade. As the strain seems morphologically quite similar to the original description of “*G*.” *amphinema* (Larsen and Patterson 1990), it was decided to use it as a basionym for *T. amphinema* n. comb.

The strain HFCC157 *Poseidogoniomonas* sp. originated from the Azores Islands. It clusters together with four sequences from the Mediterranean Sea (that were added for higher resolution) and one from the study of Phanprasert et al. (2025), *P. duplex* n. comb., that we designated as the type for this genus. The sequences differ in 7 nucleotides, mainly coming from a small insert that only HFCC 157 possesses (total length of alignment—2188 nucleotides). The two new strains share several morphological characteristics, such as cell size and flagella appendages, but seem to differ in the number of periplast plates.

The sixth marine clade consists of a mixture of sequences either traditionally assigned as “*G*.” (aff.) *amphinema* or “ *G. pacifica* ”. Both species had been morphologically well described in several publications (Al‐Qassab et al. 2002; Ekelund and Patterson 1997; Larsen and Patterson 1990; Martin‐Cereceda et al. 2010; Patterson and Lee 2000; Vørs 1993). Although our genetic analyses revealed that this clade exhibits a large genetic diversity, its members shared a high degree of morphological similarity as well as a high degree of identity in the synapomorphy motifs, where only one nucleotide difference could be detected. By defining “*G*. aff. *amphinema*” and the associated sequence EU047707 as *C. martincerecedai* n. comb. as the type species, we tried to establish a solid foundation for the taxonomy. The comprehensive species description by Martin‐Cereceda et al. (2010) serves as an ideal reference for comparing this species with others. “ *G. pacifica* ”, unfortunately, lacks ultrastructural descriptions. Within our phylogenetic analysis, two sequences were assigned to “*G*.” *pacifica*. With a pairwise distance of 0.12%, one of these sequences (LC647566) shows only minor pairwise differences to *C. martincerecedai* n. comb. (EU047707), and it is likely that it has been falsely assigned. Consequently, we chose to redefine the remaining sequence (AF508277) as a reference for *Cosmogoniomonas pacifica* n. comb. as it originates from Australia, and was sampled from the Pacific Ocean.

Our study, like other studies before, demonstrates the large diversity within the genus *Goniomonas* and offers a solution by splitting the genus into several individual genera. Still, we are aware that this might not be the last taxonomic revision that will be made for goniomonads. The large intraspecific differences hint at a much larger diversity and, by adding more sequences, may allow an even finer resolution (and splitting) either on the genus or species level. As already mentioned, it is hard to draw a line on the basis of genetic differences, especially when the morphological bauplan is similar. Whereas differences within the marine genera ranged around 4%, they were even higher within freshwater genera such as *Aquagoniomonas* n. g.

As with many protist groups, the diversity of goniomonads is still largely unknown, but, particularly with the help of modern sequencing techniques, we are slowly taking steps to get an idea about their diversity. Nevertheless, modern taxonomy, which complements molecular databases, is the backbone for all metabarcoding studies. It is crucial to keep the taxonomy up to date; otherwise much of the diversity will be lost or remain cryptic. As more and more sequences are generated, it is important to critically examine previous taxonomic decisions and revise them if necessary.

## 5 Taxonomic Summary

Assignment: Eukaryota, Cryptista, Cryptophyta, Goniomonadea, Goniomonadida

## 6 Taxonomy of Novel Genera and Species

***Goniomonas*** Stein 1878, **n. emend. Sachs et Arndt**

**Diagnosis**. A free‐living, colorless, bi‐flagellated heterotrophic cryptophyte only found in freshwater habitats, bacterivorous. Cells are laterally compressed, slightly longer than wide, and ovoid‐to‐pear shaped. The anterior end is slightly angled, posterior end is rounded. Cells carry two flagella of subequal length that can carry fine hairs or appendages. The cell body is covered by irregularly shaped, elongated periplast plates.

**Type species**. *G. truncata*

***G. truncata*** Stein 1878, **emend. Sachs et Arndt**

**Diagnosis**. Free‐living, colorless, bi‐flagellated cryptophyte from a freshwater environment. Laterally compressed cells, slightly elongated, ovoid to pear‐shaped cell body, rounded towards the posterior end, tapered, angularly truncated anterior end, with 5.9–9.3 μm length (average 7.3 μm, SD = 1.0, *n* = 22) and 4.4–7.1 μm width (average 5.5 μm, SD = 0.6, *n* = 22) of cell body. Two flagella of subequal length arise from an anterior groove and are both directed anterior‐laterally, with the anterior flagellum being 4.5–6.3 μm (average 5.4, SD = 0.6, *n* = 17) in length and the posterior flagellum being 4.0–5.6 μm (average 4.9, SD = 0.5, *n* = 16) long. Both flagella are equipped with fine hairs; the shorter flagellum carries additional spiky appendages. Cells carry five irregularly shaped periplast plates on the left side and six on the right side. A band of ejectosomes is visible on the anterior margin of the cell, with a length of 1.7–2.8 μm (average 2.3, SD = 0.4, *n* = 7) in length. Between the junctions of the periplast plates, small ejectosomes arise as globule‐like structures. Additionally, species of the genus *Goniomonas* display several unique motifs within sequences of the 18S rDNA (Figure 7) that are distinct from other genera.

**Neotype location**. Strain HFCC235 was obtained from the River Rhine, Cologne, 50°49′34″ N 6°35′34″ E

**Neotype material**. The type culture is currently deposited in the Heterotrophic Flagellate Collection Cologne, as strain HFCC235, specimen shown in Figure 1A. SEM filters are deposited at the Biology Centre of the Museum of Natural History in Upper Austria, Linz, at number OLML 2025/2675. Deep‐frozen material is deposited at the Heterotrophic Flagellate Culture Collection Cologne.

**Gene sequence data**. The 18S rDNA sequence has been deposited at GenBank with the Accession Number PX132651

**ZooBank ID**. urn:lsid:zoobank.org:act:9BF07E87‐8545‐465C‐8F5A‐BEEAE8E6AA17

***G. rhenensis* n. sp. Sachs, Nitsche et Arndt**

**Diagnosis**. Free‐living, colorless, bi‐flagellated cryptophyte from a freshwater environment. Laterally compressed cells, slightly elongated, ovoid to pear‐shaped cell body, rounded towards the posterior end, tapered, angularly truncated anterior end, with 7.8–10.8 μm (average 9.3 μm, SD = 0.4, *n* = 20) length and 5.3–7.9 μm (average 6.4 μm, SD = 0.8, *n* = 20) width of cell body. Two flagella of subequal length arise from an anterior groove and are directed anterior‐laterally, with the anterior flagellum of 3.9–6.7 μm (average 5.8 μm, SD = 0.7, *n* = 20) in length and the posterior flagellum being 3.2–5.5 μm (average 4.6 μm, SD = 0.5, *n* = 20) long. Cells carry six irregularly shaped periplast plates on the right side and four on the left side. A slim band of ejectosomes is visible on the anterior margin of the cell, with 1.6–1.9 μm (average 1.7 μm, SD = 0.1, *n* = 6) in length. Small ejectosomes can be seen between the junctions of the periplast plates.

**Type location**. Strain HFCC251 was obtained from the River Rhine, Cologne, 50°49′34″ N 6°35′34″ E

**Type material**. The type culture is currently deposited in the Heterotrophic Flagellate Collection Cologne, as strain HFCC251, specimen shown in Figure 1K. SEM filters are deposited at the Biology Centre of the Museum of Natural History in Upper Austria, Linz, at number OLML 2025/2676. Deep‐frozen material is deposited at the Heterotrophic Flagellate Culture Collection Cologne.

**Gene sequence data**. The 18S rDNA sequence has been deposited at GenBank with the Accession Number PX132652

**ZooBank ID**. urn:lsid:zoobank.org:act:F10FC759‐622B‐4F50‐A48E‐1B9AA479BD83

**Etymology:** The species name (lat. “from the Rhine”) is based on the type locality of the strain, the River Rhine.

***Aquagoniomonas* n. g. Sachs et Arndt**

**Diagnosis**. A free‐living, colorless, bi‐flagellated heterotrophic cryptophyte from freshwater habitats, bacterivorous. The cell body is laterally compressed, elongated, and leans to slightly ovoid in shape. The posterior end is slightly tapered and rounded, while the proximal end is truncated and angled. Cells carry two smooth flagella of subequal length; the anterior flagellum is slightly longer than the posterior, both being directed anterior‐laterally. The cell body is covered with irregularly shaped, elongated periplast plates, four on the left side and five on the right side, with small ejectosomes arising from in between the junctions. A band of ejectosomes can be seen transversally at the anterior margin. Additionally, species of the genus *Aquagoniomonas* display several unique motifs within sequences of the 18S rDNA (Figure 7) that are distinct from other genera.

**Type species**. *A. mylnikovii* n. g. et n. sp.

**Etymology**. The genus name refers to the origin of the strains in this genus, the freshwater environment (lat. “aqua”—water)

***A. mylnikovii* n. sp. Sachs et Arndt**

**Diagnosis**. Free‐living, colorless, bi‐flagellated cryptophyte from a freshwater environment. Laterally compressed cells, elongated, lean cell body, rounded, slightly tapered towards the posterior end, angularly truncated anterior end, with 6.1–9.7 μm (average 7.7 μm, SD = 1.1, *n* = 19) length and 4.0–5.6 μm (average 4.7, SD = 0.5, *n* = 20) width of cell body. Two flagella of subequal length arise from an anterior groove and are directed anterior‐laterally, with the anterior flagellum being slightly longer, with a length of 3.8–6.7 μm (average 5.5 μm, SD = 0.8, *n* = 8) and the posterior flagellum being 2.0–5.5 μm (average 4.0 μm, SD = 1.2, *n* = 8) long. Cells carry four irregularly shaped periplast plates on the left side and five on the right side. A prominent band of ejectosomes is visible on the anterior margin of the cell, with 1.8–2.5 μm (average 2.0 μm, SD = 0.2, *n* = 8) in length, as well as small ejectosomes that arise from in between the junctions of the periplast plates.

**Type location**. Pond, Borok, near Jaroslawl, Russia, 58°03′27″ N 38°15′37″ E

**Type material**. The type culture is currently deposited in the Heterotrophic Flagellate Collection Cologne as strain HFCC 23, the specimen shown in Figure 1N–O. SEM filters are deposited at the Biology Centre of the Museum of Natural History in Upper Austria, Linz, at number OLML 2025/2677. Deep‐frozen material is deposited at the Heterotrophic Flagellate Culture Collection Cologne.

**Gene sequence data**. The 18S rDNA sequence has been deposited at GenBank with the Accession Number PX132649

**ZooBank ID**. urn:lsid:zoobank.org:act:5614F26B‐0413‐4AB2‐BB65‐EB527140C0B6

**Etymology**. This species is dedicated to and in remembrance of Alexander P. Mylnikov for his unparalleled contributions to protozoological research. He isolated this strain 30 years ago.

***A. atacamiensis* n. sp. Sachs, Nitsche et Arndt**

**Diagnosis:** Free‐living, colorless, bi‐flagellated cryptophyte from a freshwater environment. Laterally compressed cells, slightly elongated ovoid cell body, rounded towards the posterior end, angularly truncated anterior end, with 6.0–9.0 μm (average 7.7 ± 0.8 μm; *n* = 15) length and 3.9–5.9 μm (average 5.0 ± 0.6 μm, *n* = 15) width of cell body. Two flagella of subequal length arise from an anterior groove and are directed anterior‐laterally, with the anterior flagellum being slightly longer at 7.5–7.6 μm length (average 7.6 ± 0.1, *n* = 3) than the posterior flagellum, which is 5.6 μm (average 5.6 ± 0.0 μm, *n* = 3) long. Cells carry four irregularly shaped periplast plates on the left side and five on the right side. A band of ejectosomes following the angle of the anterior margin is visible and 1.3–2.4 μm (average 2.0 ± 0.5 μm, *n* = 4) in length, as well as small ejectosomes that arise between the borders of the periplast plates.

**Type location**. Atacama, Rio Loa mouth, Chile, 21°25′41″ S, 70°03′15″ W

**Type material**. The type culture is currently deposited in the Heterotrophic Flagellate Collection Cologne as strain HFCC5007, the specimen shown in Figure 1T. SEM filters are deposited at the Biology Centre of the Museum of Natural History in Upper Austria, Linz, at number OLML 2025/2678. Deep‐frozen specimens are deposited at the Heterotrophic Flagellate Culture Collection Cologne.

**Gene sequence data**. The 18S rDNA sequence has been deposited at GenBank with the Accession Number PX132654

**ZooBank ID**. urn:lsid:zoobank.org:act:3F1E3B1E‐99CF‐40C7‐92FC‐4080B36708A9

**Etymology:** The species name refers to the geographic origin of the strain. The Rio Loa runs over 400 km through the Atacama Desert until it disembogues into the Pacific Ocean and is dedicated to the friendly people of the Atacama region.

***Limnogoniomonas* n. g. Sachs et Arndt**

**Diagnosis**. Non‐photosynthetic, colorless, single‐celled, bacterivorous cryptophycean biflagellate from freshwater habitats. Cell body laterally compressed, clearly elongated, lean shape. The posterior end is slightly rounded, proximal end is truncated, obtuse, and slightly angled. Cells carry two smooth flagella of subequal length, anterior flagellum slightly longer than the posterior, both directed anterior‐laterally. The cell body is covered with irregularly shaped, elongated periplast plates, four on the left side and five on the right side. In between those plates, small ejectosomes can be seen between the junctions of the periplast plates. At the anterior margin of the cell, a more prominent band of ejectosomes appears. Additionally, species of the genus *Limnogoniomonas* display several unique motifs within sequences of the 18S rDNA (Figure 7) that are distinct from other genera.

**Type species**. *L. fijiensis* n. g. et n. sp.

**Etymology**. The genus name is derived from the fact that the clade of *Limnogoniomonas* consists of sequences only originating from freshwater samples.

**Species included**. *L. fijiensis* n. g. et n. sp.

***L. fijiensis* n. sp. Sachs et Arndt**

**Diagnosis**. Free‐living, colorless, bi‐flagellated cryptophyte from a freshwater environment. Laterally compressed cells, elongated cell body, rounded towards the posterior end, angularly truncated and obtuse anterior end, with 7.6–11.5 μm (average 9.3 ± 1.1 μm, *n* = 20) length and 3.9–7.3 μm (average 5.4 ± 0.9 μm, *n* = 20) width of cell body. Two flagella of subequal length arise from an anterior groove and are directed anterior‐laterally, with the anterior flagellum being slightly longer at 6.0–7.7 μm (average 6.8 ± 1.1 μm, *n* = 14) length than the posterior flagellum, which is 4.5–6.6 μm (average 5.8 ± 0.5 μm, *n* = 13) long. Cells carry four irregularly shaped periplast plates on the left side and five on the right side. A band of ejectosomes following the angle of the anterior margin is visible at 1.7–2.7 μm (average 2.2 ± 0.4 μm, *n* = 7) in length. Smaller, nodule‐like ejectosomes arise in between the periplast plates.

**Type location**. Rainforest pond, Suva, Island of Fiji, 18°08′29″ S, 178°26′29″ E

**Type material**. The type culture is currently deposited in the Heterotrophic Flagellate Collection Cologne as strain HFCC162, the specimen shown in Figure 3B. SEM filters are deposited at the Biology Centre of the Museum of Natural History in Upper Austria, Linz, at number OLML 2025/2679. Deep‐frozen specimens are deposited at the Heterotrophic Flagellate Culture Collection Cologne.

**Gene sequence data**. The 18S rDNA sequence has been deposited at GenBank with the Accession Number PX132650

**ZooBank ID**. urn:lsid:zoobank.org:act:DE170391‐7655‐4DA9‐B7EF‐7F4C06340482

**Etymology**. The species name refers to the geographical origin of the type species, the Fiji Islands in the Pacific Ocean, and is dedicated to the friendly people of the Fiji archipelago.

***Baltigoniomonas* n. g. Sachs et Arndt**

**Diagnosis**. A non‐photosynthetic, colorless, single‐celled, bacterivorous cryptophycean bi‐flagellate from marine or brackish habitats. Cell body rounded, ovoid in shape, with a rounded posterior end and a truncated anterior end. Cells have four periplast plates on the left side and three on the right side. Two flagella of subequal length, slightly longer than cell length and with different orientations. During movement, the anterior flagellum is directed anterior‐laterally; the posterior flagellum is directed towards the cell. The anterior flagellum is equipped with hairs; the posterior flagellum is smooth. Ejectosomes can be seen arising between the junctions of the periplast plates. Species of the genus *Baltigoniomonas* display several unique motifs within sequences of the 18S rDNA (Figure 7) that are distinct from other genera.

**Type species**. *B. juergensii* n. sp. Sachs et Arndt

**Etymology**. The prefix “*Balti*” refers to the Baltic Sea, where the type species of this genus was sampled.

***B. juergensii* n. sp. Sachs et Arndt**

**Diagnosis**. Free‐living, colorless, bi‐flagellated cryptophyte from a brackish environment. Laterally compressed, ovoid cells with a truncated anterior end, 5.9–9.3 μm (average 7.7 ± 0.9 μm, *n* = 20) long and is 4.8–7.1 μm (average 6.1 ± 0.6 μm, *n* = 20) wide. Flagella are of unequal length; the anterior flagellum is directed anterior‐laterally and 4.9–7.6 μm (average 6.7 μm, SD = 0.6, *n* = 20) long and is equipped with mastigonemes. The posterior flagellum is directed towards the cell body and oftentimes carried laying over the cell and is 5.5–9.0 μm (average 7.2 ± 0.9 μm, *n* = 20) in length and smooth. Cells carry four rectangular periplast plates on the left side and three on the right side. No band of ejectosomes could be observed at the anterior margin of the cell, but smaller nodule‐like ejectosomes were between the periplast plates.

**Type location**. Island Hiddensee, Germany, Baltic Sea, 54°35′37″ N 13°06′34″ E

**Type material**. The type culture is currently deposited in the Heterotrophic Flagellate Collection Cologne as strain HFCC22, the specimen shown in Figure 4D. SEM filters are deposited at the Biology Centre of the Museum of Natural History in Upper Austria, Linz, at number OLML 2025/2680. Deep‐frozen specimens are deposited at the Heterotrophic Flagellate Culture Collection Cologne.

**Gene sequence data**. The 18S rDNA sequence has been deposited at GenBank with the Accession Number PX132648

**ZooBank ID**. urn:lsid:zoobank.org:act:9343D2FC‐F9BB‐420A‐A975‐41FDB724F413

**Etymology**. The species is dedicated to Klaus Jürgens for his major contributions to the research on microbes of the Baltic Sea.

***Neptunogoniomonas* n. g. Sachs et Arndt**

**Diagnosis:** “A colorless, bacterivorous marine flagellate with a flattened cell body. Cell 8–11 μm long, 6–7 μm wide, somewhat truncated at the anterior. Two subequal flagella are slightly longer than the cell and arise from an anterior depression. One flagellum is directed forward, whereas the other runs backward towards the cell posterior through a longitudinal groove. A conspicuous band of large ejectosomes runs across the anterior margin. Younger cells tend to have a more pointed posterior end compared to older cells, which have a more rounded end. When swimming, the cell rotates around its longitudinal axis. Swimming cells slide along surfaces with the posterior flagellum trailing beneath the cell.” (Kim and Archibald 2013). Small ejectosomes arise between the junctions of the periplast plates. Additionally, species of the genus *Neptunogoniomonas* display several unique motifs within sequences of the 18S rDNA (Figure 7) that are distinct from other genera.

**Type species:** *N. avonlea* (Kim and Archibald 2013) n. comb. Sachs et Arndt

**Etymology:** The prefix Neptuno refers to the Roman god of the oceans, with reference to the Atlantic Ocean where the type species of the genus has been sampled.

***Marigoniomonas* n. g. Sachs et Arndt**

**Diagnosis**. Non‐photosynthetic, colorless, single‐celled, bacterivorous cryptophycean biflagellate from marine environments. Cells are laterally compressed and roundish to D‐shaped due to a rounded posterior end and truncated, angled anterior end, with the angle pointing upwards from the attachment point of the two unequally long flagella, whereas the shorter flagellum is directed anterior‐laterally and the longer is directed towards the cell body. Cells carry three to five periplast plates on the left side and three to four on the right side, with small ejectosomes arising in between them. A band of ejectosomes can be seen at the anterior margin of the cell.

**Type species**. *Marigoniomonas lingua* (Phanprasert et al. 2025) n. comb. Sachs et Arndt

**Etymology**. The genus name refers to the marine environment as an origin for all included taxa.

***M. lingua* (**Phanprasert et al. 2025 **) n. comb. Sachs et Arndt**

**Basionym: *Goniomonas lingua*** Phanprasert et al. 2025

For diagnosis of the original description of *M. lingua* n. comb. see Phanprasert et al. (2025).

***M. ulleungensis* (**Phanprasert et al. 2025**) n. comb. Sachs et Arndt**

**Basionym:** *Goniomonas ulleungensis* Phanprasert et al. 2025

For diagnosis of the original description *M. ulleungensis* n. comb. see Phanprasert et al. (2025)

***Thalassogoniomonas* n. g. Sachs et Arndt**

**Diagnosis**. Non‐photosynthetic, colorless, single‐celled, bacterivorous cryptophycean biflagellate from marine environments. Cells are cup‐shaped, with a tapered posterior end and a truncated, yet rounded, anterior end. Two unequally long flagella arise from the anterior margin of the cell; the anterior flagellum is anterior‐laterally directed, and the posterior flagellum is carried over the cell in an almost curled manner. A prominent band of ejectosomes is visible transversally at the anterior margin of the cell. Species of the genus *Thalassogoniomonas* display several unique motifs within sequences of the 18S rDNA (Figure 7) that are distinct from other genera.

**Type species**. *T. amphinema* n. comb.

**Etymology**. The prefix'Thalasso'(Greek: thalassa—ocean) refers to the ocean as the environment of origin.

***T. amphinema* (**Larsen and Patterson 1990**) n. comb. Sachs et Arndt**

**Basionym:** *Goniomonas amphinema* Larsen and Patterson 1990

**Diagnosis**. Free‐living, colorless, bi‐flagellated cryptophyte from the marine environment. Laterally compressed cells, cup‐shaped, slightly elongated, with a tapered posterior and truncated, obtuse anterior end; cells of 4.3–5.7 μm (average 5.2 ± 0.5 μm, *n* = 19) length and 3.1–4.6 μm (average 3.8 ± 0.5 μm, *n* = 19) width of cell body. Two flagella of unequal length, the anterior flagellum with 3.3–6.3 μm (average 5.1 ± 0.7 μm, *n* = 16) and directed anterior‐laterally; and the posterior flagellum generally directed towards the cell body, 5.2–8.6 μm (average 7.4 ± 0.8 μm, *n* = 18) long. A prominent band of ejectosomes is visible on the anterior margin of the cell with 1.7–2.3 μm (average 1.9 ± 0.2 μm, *n* = 12) length.

**Neotype location**. North Sea, shore of the Island Helgoland (Germany), 54°11′01″ N 7°53′27″ W from a depth of 950 m.

**Neotype material**. The neotype culture is currently deposited in the Heterotrophic Flagellate Collection Cologne as strain HFCC1666, the specimen shown in Figure 4N. Deep‐frozen specimens are deposited at the Heterotrophic Flagellate Culture Collection Cologne.

**Gene sequence data**. The 18S rDNA sequence has been deposited at GenBank with the Accession Number PX132653

**ZooBank ID**. urn:lsid:zoobank.org:act:C8EF8E32‐A211‐48A9‐9B2A‐AE69660701B3

**Remarks:** Since the light microscopic images of *G. amphinema* (Larsen and Patterson 1990), cell size and width, as well as the length‐to‐width ratio appear to be very similar to HFCC1666, it is reasonable to designate *T. amphinema* as the neotype for *Goniomonas amphinema*.

***Poseidogoniomonas* n. g. Sachs et Arndt**

**Diagnosis**. Non‐photosynthetic, colorless, single‐celled, bacterivorous cryptophycean biflagellate from marine environments. Cells are laterally compressed, with a roundish, slightly elongated cell body and sides almost parallel to each other. Cells carry three to five periplast plates on the right side and four to five on the left side. Two unequal flagella with different directions: a smooth, shorter anterior flagellum directed anterior‐laterally, and a longer posterior flagellum directed towards or curved over the cell, equipped with fine hairs. Ejectosomes appear as a transversal band as well as small globules in between the periplast plates. Species of the genus *Poseidogoniomonas* display several unique motifs within sequences of the 18S rDNA (Figure 7) that are distinct from other genera.

**Type species**. *Poseidogoniomonas duplex* n. comb.

**Etymology**. The addition “*Poseido*” refers to the Greek word for god of the ocean, Poseidon, and points to the marine origin of the type strain.

***Poseidogoniomonas duplex* (**Phanprasert et al. 2025**) n. comb. Sachs et Arndt**

**Basionym:** *Goniomonas duplex* Phanprasert et al. 2025

For diagnosis of the original description of *Poseidogoniomonas duplex* n. comb. see Phanprasert et al. (2025).

***Cosmogoniomonas* n. g. Sachs et Arndt**

**Diagnosis**. Non‐photosynthetic, colorless, single‐celled, bacterivorous cryptophycean biflagellate from marine environments. Cells are ovate with a truncated anterior and rounded posterior end, dorso‐ventrally flattened. Two flagella of similar length, one pointing forward (can be equipped with appendages), one bent ventrally, trailing. Cells are covered with periplast plates (exact number not stated, see remarks). Between the plates, lines of ejectosomes can be found in the ridges. An additional transversal band of ejectosomes can be seen at the anterior end of the cell. Species of the genus *Cosmogoniomonas* display several unique motifs within sequences of the 18S rDNA (Figure 7) that are distinct from other genera.

**Type species:** *C. martincerecedai* (Martin‐Cereceda et al. 2010) n. comb. Sachs et Arndt

**Etymology:** “*Cosmo*” refers to the supposedly cosmopolitan distribution of this genus.

**Remarks:** Species within the genus *Cosmogoniomonas* n. g. are morphologically relatively similar but genetically relatively diverse within a significantly separated clade (Figure 5). Since morphological features are well described, we here define the 18S rDNA genotype EU047707 (Martin‐Cereceda et al. 2010) as the neotype sequence for *C. martincerecedai* n. comb. The detailed description by Martin‐Cereceda et al. (2010) gives the best reference for this species. For “ *G. pacifica*, *”* light microscopic studies exist (Larsen and Patterson 1990). In GenBank, there are only two sequences at the moment that were assigned to “*G. pacifica”*. One sequence (LC647566) only has a minor (0.12%) pairwise difference to *C. martincerecedai* n. comb. (EU047707). Hence, we have decided to define the other existing sequence (AF508277, Deane et al. 2002) as the neotype sequence for *C. pacifica* n. comb., as it includes the description of Larsen and Patterson (1990) in the description for this species. Our choice is underpinned by the fact that AF508277 originates from the Australian Pacific.

***C. martincerecedai* (**Martin‐Cereceda et al. 2010**) n. comb. Sachs et Arndt**

**Diagnosis adapted from** Martin‐Cereceda et al. (2010): Free‐living, colorless, bi‐flagellated cryptophyte from the marine environment. Cells have an anterior truncate oval shape and are 5.6 ± 0.51 μm long and 3.5 ± 0.57 μm wide (*n* = 90). One straight shorter flagellum beats jerkily in all directions (5.18 ± 0.44 μm long), more rapidly than the trailing, longer one (6.32 ± 0.45 μm long, *n* = 45). The short flagellum carries flagellar appendages. The flagellar vestibulum is about one‐fourth of the cell length. A line of ejectosomes appears transversally in the anterior end of the cell. A cytopharynx structure is often visible along the longitudinal axis of the cell. Between the periplast plates, rows of emerging ejectosomes are visible. The central part of the anterior part of the cell appears raised. On the ventral anterior side, a granular area is visible.

**Type location**. Menai Strait, North Wales, UK, 53.231 N, 4.164 W

**Type material**. Culture collection of Algae and Protozoa, CCAP 980/2, Figure 5 in Martin‐Cereceda et al. (2010).

**Gene sequence data**. The 18S rDNA sequence has been deposited at GenBank with the Accession Number EU047707 (Martin‐Cereceda et al. 2010).

***C. pacifica* (**Larsen and Patterson 1990**) n. comb. Sachs et Arndt**

**Basionym:** *G. pacifica* Larsen and Patterson (1990)

**Diagnosis of the original description by** Larsen and Patterson (1990): “Cell outline ovate, truncate anteriorly, rounded posteriorly, 8‐10 μm long, 6‐8 m wide, laterally compressed. With a transverse band of ejectosomes near the anterior end. The left‐hand side of the cell has 2–3 longitudinal, very delicate ridges, the right‐hand side has 3 ridges which are usually more distinct. The nucleus situated in the middle of the cell near the dorsal side. Two equal or slightly unequal (dorsal longer) flagella emerge from a small depression anteriorly near the dorsal surface, about 3/4 the cell length. At their insertion the bases lie parallel to the axis of the cell. When swimming, the flagella diverge; the one situated closest to the dorsal surface points forwards and bends only slightly in the dorsal direction, while the other flagellum usually bends towards the ventral side. The cell skids parallel to the substratum when moving.”

**Type location**. Port Phillip Bay, Melbourne, Victoria, Australia.

**Type material**. Figure 1g in Larsen and Patterson (1990).

**Gene sequence data**. The 18S rDNA sequence has been deposited at GenBank with the Accession Number AF508277 (Deane et al. 2002).

**Remarks**. Deane et al. (2002) have identified their strain as “*Goniomonas” pacifica*. Since this strain was isolated in the region from where the originally described “*Goniomonas” pacifica* also originated, we decided to assign the sequence provided by Deane et al. (2002) to the species description. The culture of this species is deposited at the Provasoli‐Guillard Center for Culture of Marine Phytoplankton, strain CCMP1869.

## Author Contributions

Maria Sachs: data curation, formal analysis, investigation, visualization, writing – original draft. Frank Nitsche: investigation, supervision, writing – review and editing. Hartmut Arndt: conceptualization, investigation, supervision, funding acquisition, project administration, writing – review and editing.

## Supporting information


**Data S1:** Supporting Information.

**Video S1:** jeu70038‐sup‐0002‐VideoS1.mp4.

**Video S2:** jeu70038‐sup‐0003‐VideoS2.mp4.

**Video S3:** jeu70038‐sup‐0004‐VideoS3.mp4.

**Video S4:** jeu70038‐sup‐0005‐VideoS4.mp4.

**Video S5:** jeu70038‐sup‐0006‐VideoS5.mp4.

## Data Availability

Data are published or will be made available on request.
