# Supplementary material for: Cryptic Cryptophytes—Revision of the Genus Goniomonas
Source: J Eukaryot Microbiol. 2025 Sep 12;72(5):e70038. doi: 10.1111/jeu.70038 (PMC12432292; doi:10.1111/jeu.70038)
Supplement: Supplementary file 1 — Data S1: Supporting Information. [file JEU-72-e70038-s006.zip › Supplementary material.pdf]

Supplementary Material

Cryptic Cryptophytes – revision of the genus *Goniomonas*

Maria Sachs[1, 2]**, Frank Nitsche**[1] **and Hartmut Arndt**[1]*

[1]University of Cologne. Institute of Zoology. Department of General Ecology and Limnology. Zuelpicher Straße 47b. 50674 Cologne. Germany
[2]Senckenberg am Meer, DZMB – German Centre for Marine Biodiversity Research, Südstrand 44, 26382 Wilhelmshaven, Germany.

Table 1 List of chemicals and their amounts to produce one litre of Schmalz-Pratt Medium with 23 ‰ salinity.

Chemical component	Amount [g/l] for 23 PSU
NaCl	18.77
KCl	0.45
$MgCl_2$ x 6 H_2O	3.67
$MgSO_4$ x 7 H_2O	4.61
$CaCl_2$ x 2 H_2O	0.97
KNO_3	0.1
K_2HPO_4 x 3 H_2O	0.01

Table 2 Pairwise distances (in percent) of 18S rDNA sequences for freshwater clusters including the genera *Goniomonas* and *Aquagoniomonas* n. g.

	Strain	1	2	3	4	5	6	7	8	9
1	HFCC 235 *Goniomonas truncata*	0								
2	HFCC 232 *Goniomonas truncata*	0	0							
3	HFCC 251 *Goniomonas rhenensis* n. sp.	3.83	3.83	0						
4	HFCC 23 *Aquagoniomonas mylnikovii* n. g. et. n. sp.	10.47	10.47	12.65	0					
5	LC000677	10.49	10.49	12.55	0.47	0				
6	HFCC 163 "*Aquagoniomonas* sp."	10.77	10.77	12.75	0.61	0.52	0			
7	HFCC 5007 *Aquagoniomonas atacamiensis* n. g. et n. sp.	10.4	10.4	12.69	3.1	0.82	0.87	0		
8	HFCC 5008 *Aquagoniomonas* sp.	9.86	9.86	12.91	0.72	0.82	0.78	0.61	0	
9	AY360459	10.71	10.71	13.11	0.98	0.51	1.03	1.42	1.32	0

Table 3 Pairwise distances in percent based on 18S rDNA for the cluster *Limnogoniomonas* n. g.

	Strain	1	2	3	4
1	HFCC 162 *Limnogoniomonas fijiensis* n. g. et n. sp.	0			
2	DQ980479	0.17	0		
3	U03072	0.28	0.42	0	
4	HQ659565	7.7	8.22	7.98	0

Table 4 Pairwise distances (in percent) of 18S sequences for the cluster including the genus *Baltigoniomonas* n. g.

	Strain	1	2
1	HFCC 22 *Baltigoniomonas juergensii* n. g. et n. sp.	0	
2	MK177625	0.31	0

Table 5 Pairwise distances (in percent) of 18S sequences for the cluster including the genus *Neptunogoniomonas* n. g.

	Strain	1	2	3
1	HFCC 1525	0		
2	JQ434475 *Neptunogoniomonas avonlea* n. comb.	0.31	0	
3	LC647565	0	0.41	0

Table 6 Pairwise distances based on 18S rDNA for the cluster *Cosmogoniomonas* n. g.

	Strain	1	2	3	4
1	HFCC 662 *Cosmogoniomonas* sp.	0			
2	LC674566	0.13	0		
3	EU047707 *Cosmogoniomonas martincerecedai* n. comb.	0.13	0.12	0	
4	AF508277 *Cosmogoniomonas pacifica* n. g. et n. sp.	3	2.57	2.57	0

Table 7 Pairwise distances in percent based on 18S rDNA sequences for the genera *Marigoniomonas* n. g., *Thalassogoniomonas* n. g. and *Poseidogoniomonas* n. g. and the associated sequences.

	Strain	1	2	3	4	5	6	7	8
1	HFCC 841 *Marigoniomonas* sp.	0							
2	KX431494	2.12	0						
3	HFCC 5003 *Marigoniomonas* sp.	0.26	2.21	0					
4	HFCC 272 *Marigoniomonas* sp.	3.4	3	3.53	0				
5	HFCC 1666 *Thalassogoniomonas amphinema* n. g. et n. sp.	6.1	4.76	6.32	6.78	0			
6	AY360454	6	4.22	6.18	6.38	1.44	0		
7	HFCC 157 *Poseidogoniomonas* sp.	6.09	3.87	6.27	6.53	3.73	3.33	0	
8	BEAP0122b	6.38	4.31	6.73	7.52	4.67	3.85	3.38	0

Table 8 List of strains isolated and described in the present study. The number of the strains in the Heterotrophic Flagellate Culture Collection Cologne (HFCC), the location and the geographic coordinates of the sampling sites, the habitat and the NCBI accession number of the 18S rDNA sequence are given.

Species	Isolate (HFCC)	Location of isolation	Latitude/Longitude	Habitat	Accession number (NCBI)
Baltigoniomonas juergensii n. g. et n. sp.	22	Kloster, Island Hiddensee, Germany, Baltic Sea	54°35'37"N/ 13°06'34"E	Brackish water, 8 PSU, muddy sand	XXX
Marigoniomonas sp.	841	Loberia, Atacama, Chile, Southern Pacific (Kelp, Pacific Ocean)	23°3'13,72"S/ 70°32"58,63"W	Marine, 36 PSU, shore biofilm,	XXX
Marigoniomonas sp.	272	Ribeira Brava, Madeira Island, Portugal, North Atlantic	32°36`33"N/ 16°53`16"W	Marine, deep sea, 35 PSU, soft sediment (1000m depth)	XXX
Thalassogoniomonas amphinema n. g. et n. sp.	1666	Helgoland Island, Germany, North Sea	54°11'01'' N/ 07°53'23'' E	Marine, 34 PSU, shore biofilm	XXX
Poseidogoniomonas sp.	157	Island of Faial, Azores, Portugal (Atlantic Ocean)	38°28'25"N/ 28°27'16"W	Marine, 36 PSU, fine sand (450m depth)	XXX
Goniomonas truncata	235	River Rhine, Cologne, Germany	50°49'34"N/ 6°35'34"E	Freshwater, river snow	XXX
Goniomonas rhenensis n. sp.	251	River Rhine, Cologne, Germany	50°49'34"N/ 6°35'34"E	Freshwater, river snow	XXX
Aquagoniomonas mylnikovii n. g. et n. sp.	23	Pond in Borok, Jaroslawl, Russia	58°03'27"N/ 38°15'37"E	Freshwater, pond biofilm	XXX
Aquagoniomonas atacamiensis n. g. et n. sp.	5007	Rio Loa, Atacama, Chile	21°25'41"S/ 70°03'15"W	Freshwater, river biofilm	XXX
Limnogoniomonas fijiensis n. g. et n. sp.	162	Rainforest pond, Suva, Fiji	18°08'29"S/ 178°26'29"E	Freshwater, plankton	XXX
